# Comparative Efficacy and Safety of Advanced Intravitreal Therapeutic Agents for Noninfectious Uveitis: A Systematic Review and Network Meta-Analysis

**DOI:** 10.3389/fphar.2022.749312

**Published:** 2022-04-05

**Authors:** Weiting Liao, Zhenyu Zhong, Guannan Su, Xiaojie Feng, Peizeng Yang

**Affiliations:** The First Affiliated Hospital of Chongqing Medical University, Chongqing Key Lab of Ophthalmology, Chongqing Eye Institute, Chongqing Branch of National Clinical Research Center for Ocular Diseases, Chongqing, China

**Keywords:** drug implants, intravitreal agents, drug delivery system (DDS), steroid, anti-VEGF (vascular endothelial growth factor) agents

## Abstract

**Background:** To compare the efficacy and safety of advanced intravitreal therapeutic regimens, including a dexamethasone implant at 350 and 700 μg; a fluocinolone acetonide (FA) implant, 0.2 µg/day, 0.59 and 2.1 mg; intravitreal bevacizumab, 1.25 mg; intravitreal ranibizumab, 0.5 mg; intravitreal triamcinolone acetonide (IVTA), 2 and 4 mg; and standard of care (SOC, systemic therapy) for noninfectious uveitis.

**Methods:** We searched the Cochrane Library database, EMBASE, Medline, clinicaltrials.gov until April 2021 with 13 RCTs (1806 participants) identified and conducted a pairwise and Bayesian network meta-analysis with random effects.

**Results:** No specific regimen showed a statistically significant advantage or disadvantage to another treatment regimen with regard to efficacy. However, the FA implant, 0.59 mg was associated with a higher risk of cataract (RR 4.41, 95% CI 1.51–13.13) and raise in intraocular pressure (IOP) (RR 2.53 95% CI 1.14–6.25) compared with SOC at 24 months. IVTA, 4 mg at 6 months was associated with lower risk of IOP rising compared with FA implant, 0.2 µg/day at 36 months (RR 3.43 95% CI 1.12–11.35).

**Conclusion:** No intravitreal therapeutic regimens showed a significant advantage or disadvantage with regard to efficacy. However, SOC was associated with lower risk of side effects compared with FA implants. IVTA, 4 mg, might be the best choice with lowest risk of IOP rising.

**Systematic Review Registration:** clinicaltrials.gov, identifier CRD42020172953

## Introduction

Uveitis encompasses a group of inflammatory ocular disorders, and noninfectious uveitis, in particular, is reported as one of the dominant global causes of avoidable visual impairment (Bloch-Michel and Nussenblatt, 1987; Durrani et al., 2004; de Smet et al., 2011; Hsu et al., 2019). Compared with other diseases with high blindness incidence, uveitis is more likely to cause vision loss in the working age population (de Smet et al., 2011), leading to impaired vision in up to 10% of those affected (de Smet et al., 2011; Koronis et al., 2019).

Twenty years ago, an expert panel recommended systemic steroids and immunosuppressants (systemic therapy) as the mainstay treatment for noninfectious uveitis (Jabs et al., 2000). However, due to the existence of the blood–retinal barrier, many drugs do not reach the site of inflammation (Hosoya and Tachikawa, 2009). Intraocular injection of therapeutic agents is, therefore, proposed to circumvent this problem (Hosoya and Tachikawa, 2009). Intravitreal steroid injections are also propagated to avoid the systemic side effects of steroids (Nayak and Misra, 2018; Ormaechea et al., 2019). Intravitreal steroids are currently used in the clinic and include intravitreal triamcinolone acetonide (IVTA), the dexamethasone (DEX) intravitreal implant, and the fluocinolone acetonide (FA) intravitreal implant. Retisert (FA 0.59 mg; Bausch and Lomb, Rochester, NY), Ozurdex (DEX 0.7 mg; Allergan, Inc., Irvine, CA), YUTIQ (FA 0.18 mg; EyePoint Pharmaceuticals, Inc., MA, United States), and ILUVIEN (FA 0.19 mg; Alimera Sciences, Aldershot, United Kingdom), are four major intravitreal implants used for prevention of relapse of noninfectious uveitis involving the posterior segment. Intravitreal Bevacizumab ((IVB) Avastin; Genentech, Inc., South San Francisco, CA) and intravitreal ranibizumab ((IVR) Lucentis; Novartis Pharma AG, Basel, Switzerland, and Genentech, Inc., South San Francisco, CA), the recombinant humanized antivascular endothelial growth factor (VEGF) monoclonal antibody, are studied to treat uveitic complications, such as cystoid macular edema, retinal neovascularization, and choroidal neovasularization (Gulati et al., 2011). With the introduction of new drugs and emergence of sustained-release technology, intravitreal therapy has made great progress.

Although studies compare the efficacy of some intravitreal drugs, these studies have not been comprehensive (Brady et al., 2016; Vieira et al., 2020). At this moment, there are, however, no practical clinical guidelines or systematic reviews that compare the efficacy and safety of different intravitreal therapeutic agents in noninfectious uveitis and this is, therefore, the subject of the study reported here.

Network meta-analyses is a novel method that is able to compare multiple treatment options and represents a breakthrough in meta-analysis studies that normally only discuss two-arm clinical trials (Caldwell et al., 2005; Li et al., 2011). In this study, Bayesian network meta-analysis of published research is performed to obtain relative rankings of efficacy and safety for DEX implant, 350 μg; DEX implant, 700 μg; FA implant, 0.18/0.19 mg; FA implant, 0.59 mg; FA implant, 2.1 mg; IVB, 1.25 mg; IVR, 0.5 mg; IVTA, 2 mg; IVTA, 4 mg and systemic therapy for noninfectious uveitis.

## Methods

The Preferred Reporting Items for Systematic Reviews and Meta-analyses (PRISMA) (PRISMA checklist, Supplementary Table S1) method was used for our network meta-analysis (https://www.equator-network.org/reporting-guidelines/prisma/). (Hutton et al., 2015). This study is registered with PROSPERO, number CRD42020172953.

Yutiq and Iluvien implants were almost the same, containing 0.18 and 0.19 mg FA implants, respectively. In this study, clinical evidence of 0.18 and 0.19 mg FA implants were pulled together, delivering doses of 0.2 μg per day.

### Databases and Search Strategy

The databases searched included Cochrane Library databases, EMBASE, Medline, and clinicaltrials.gov until April 2021. No date or language restrictions were set for published and unpublished studies. Supplementary Table S2 shows the detailed search strategies used. We also searched the website of the U.S. Food and Drug Administration (FDA) to identify very recently approved drugs in April 2021 (https://www.fda.gov/).

### Eligibility Criteria

Studies were eligible for our network meta-analysis if they met the following criteria: 1) population: participants with vision better than hand motion and a history of noninfectious intermediate uveitis, posterior uveitis, or panuveitis; 2) intervention: surgical injection of intravitreal therapeutic agents: DEX implant or FA implant or IVTA or IVB or IVR; 3) controls: at least one arm with sham injection or observation treatment or standard of care (SOC) or a different comparative intravitreal therapeutic regimens. For the SOC group, subjects were administrated with systemic therapy following expert panel guidelines (Jabs et al., 2000). Prednisolone or an equivalent corticosteroid alone was used, or an immunosuppressive agent was added to the therapy (Jabs et al., 2000). For the sham injection group (placebo), procedures were performed identically as in the injection group except for the intravitreal therapeutic agent administration into the vitreous cavity; 4) outcome: at least one outcome concerning efficacy or safety; 5) study design: randomized controlled trial (RCT).

### Study Selection

Two individuals (WTL, XJF) independently screened the titles and abstracts found in the various databases and identified potential eligibility by retrieving the full-text articles. The final eligibility was independently decided by these two individuals. If they disagreed on a candidate article, a third person was consulted (ZYZ).

### Data Collection and Risk of Bias Assessment

Data from selected studies were extracted by two independent individuals and included numbers of participants, interventions, study design, quantitative results of interventions, clinical endpoints, and risk of bias. In the case of missing data that could not be extracted directly from the article, we sent an email to the authors and asked for raw data. GetData GraphDigitizer (http://getdata-graph-digitizer.com) was also used to obtain digital information from figures. Cochrane Risk of Bias Tool was used to assess the quality of RCTs (Barcot et al., 2019).

### Outcomes Definition

Efficacy was the primary outcome and referred to a best-corrected visual acuity (BCVA) change from baseline, percentage of eyes achieving a vitreous haze grading of zero, uveitis recurrence rate, and retinal thickness change from baseline. Safety was a secondary outcome referring to incidence of cataract formation or progression as well as use of IOP-lowering medications after baseline.

### Data Synthesis and Analysis

A pairwise meta-analysis was first used to estimate direct comparison between two interventions. We estimated risk ratios (RRs) for dichotomous outcomes and mean deviations (MDs) for continuous variables in both pairwise meta-analysis and Bayesian network meta-analysis. A random-effects model was applied to synthesize effect sizes.

Heterogeneity, generally defined as variations in the estimated effect between studies, was estimated through Higgins I-squared inconsistency statistics (Trikalinos and Ioannidis, 2001). A large degree of heterogeneity (e.g., I^2^ > 50%) was considered as statistically significant (DerSimonian and Kacker, 2007).

All our models were fitted in a Bayesian network meta-analysis (Li et al., 2011; Higgins et al., 2012). In our study, Bayesian modeling relied on the Markov chain Monte Carlo (MCMC) methods under noninformative priors in favor of R (version 3.6.3 with JAGS) to generate Bayesian probability estimates (Lu and Ades, 2004; Ades et al., 2008; Song et al., 2012). We assume that the number of chains equals four, tuning iterations equal 20,000, and simulation iterations equal 50,000. Convergence of models was checked by observation of Brooks–Gelman–Rubin diagnostic plot (Supplementary Figure S1), trace, and density plot (Supplementary Figure S2) (Brooks and Gelman, 1998; Coleman et al., 2012; Jansen and Naci, 2013). The probabilities of the best, second, third, and so on until the least effective treatment are presented, ranging from 0% to 100%, and values closer to 100% are higher probabilities (Salanti et al., 2011).

We further assess inconsistency (the difference of estimates of effect between direct comparison and indirect comparison) of evidence in network meta-analysis using node-splitting models (Dias et al., 2010). If *p* value testing the inconsistency between direct and indirect evidence in this network meta-analysis is significant (*p* < .05), then inconsistency is denoted indicating a violation of the network analysis assumption.

Sensitivity analysis was performed by deleting studies that resulted in high heterogeneity in pairwise meta-analysis (I-squared >50%). A Bayesian network meta-analysis was repeated after omitting studies leading to high heterogeneity.

All analyses were conducted using the “gemtc” and “rjags” packages of R, version 3.6.3 (R Foundation) (Supplementary Table S3).

## Results

### Database and Study Quality

#### Study Selection

We identified 3968 records following database searching. After removing duplicates, 840 records were screened via their title and abstracts, of which 192 were further assessed for eligibility (Supplementary Figure S3). We finally included 13 RCTs (Callanan et al., 1960; Lowder et al., 1960; Pavesio et al., 2010; Soheilian et al., 2010; Kempen et al., 2011; EMC, 2012; Rahimi et al., 2012; Sangwan et al., 2015; Shin and Yu, 2015; Lai et al., 2018; Staurenghi et al., 2018; Thorne et al., 2019; Jaffe et al., 2020) for network analysis (Supplementary Tables S4, S5). The analyzed regimens include DEX implant (Ozurdex; Allergan, Inc., Irvine, CA), 700 μg; DEX implant, 350 μg; FA implant (YUTIQ; EyePoint Pharmaceuticals, Inc., MA, United States. Iluvien; Alimera Sciences, Aldershot, United Kingdom), 0.2 µg/day; FA implant (Retiser; Bausch and Lomb, Rochester, NY), 0.59 mg; FA implant, 2.1 mg; IVB, 1.25 mg (Avastin; Genentech, Inc., South San Francisco, CA); IVR, 0.5 mg (Lucentis; Novartis Pharma AG, Basel, Switzerland, and Genentech, Inc., South San Francisco, CA); IVTA, 2 mg; IVTA, 4 mg and systemic therapy for noninfectious uveitis. To the best of our knowledge, the DEX implant, 350 µg and FA implant, 2.1 mg were not commercialized.

#### Study Characteristics

In included studies, eight multicenter RCTs (61.5%) and five single-center RCTs (38.5%) were eligible for further analyses. Included studies were published between 2008 and 2020. Included studies enrolled a total of 1806 participants with sample size ranging from 21 (Staurenghi et al., 2018) to 278 (Callanan et al., 1960). The included studies had participants that were diagnosed with noninfectious intermediate uveitis, posterior uveitis, or panuveitis. Mean age of patients in all studies was around 50, ranging from 40.4 to 55.3 except for one (Rahimi et al., 2012), where patients aged 23.1 ± 11.2 years. Half of the studies had a follow-up of at least 24 months, whereas follow-up in two studies was 6 months. One study (Kempen et al., 2017) even reported visual acuity data during a time period of 7 years (Multicenter Uveitis Steroid Treatment (MUST) trial). Two studies (Lowder et al., 1960; Thorne et al., 2019) had three arms, and the rest of the studies contained two arms. Direct comparison between two different intravitreal therapeutic regimens was reported in six studies (Callanan et al., 1960; Lowder et al., 1960; Soheilian et al., 2010; Rahimi et al., 2012; Sangwan et al., 2015; Thorne et al., 2019). We also included the MINERVA (Lai et al., 2018) and PROMETHEUS (Staurenghi et al., 2018) trials, in which subgroups of noninfectious intermediate uveitis, posterior uveitis, or panuveitis were also contained in the network meta-analysis.

**FIGURE 1:**
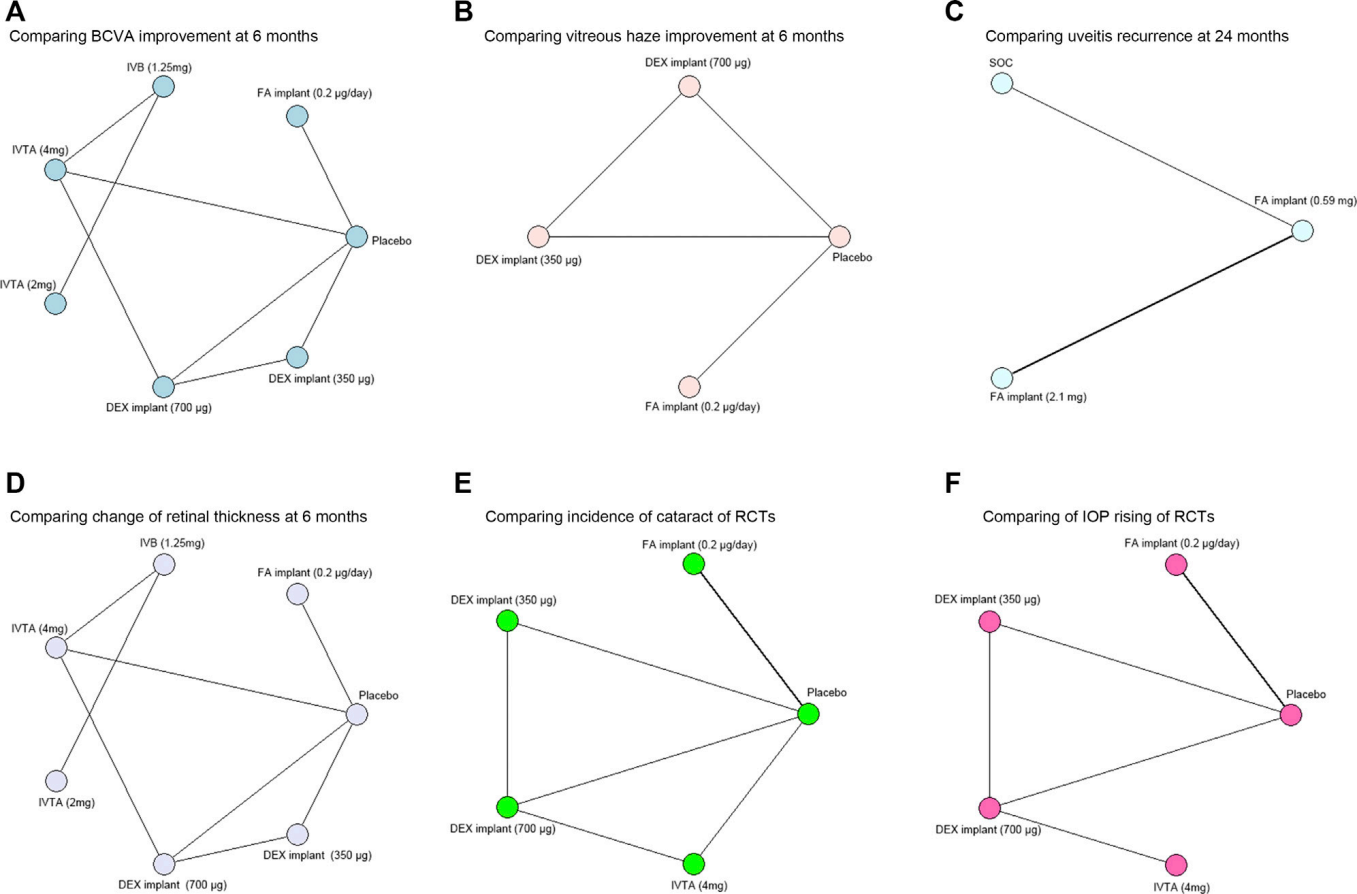
Network meta-analysis diagrams of treatment regimens in RCTs. Each node represents one intervention. The edges represent direct comparisons, and the width of the edge is proportional to the number of studies. **(A)** Network meta-analysis diagram of treatments for comparing BCVA improvement at 6 months. **(B)** Network meta-analysis diagram of treatments for comparing vitreous haze improvement at 6 months. **(C)** Network meta-analysis diagram of treatments for comparing uveitis recurrence at 24 months. **(D)** Network meta-analysis diagram of treatments for comparing change of retinal thickness at 6 months. **(E)** Network meta-analysis diagram of treatments for comparing incidence of cataract. **(F)** Network meta-analysis diagram of treatments for comparing IOP rising. BCVA, best-corrected visual acuity; DEX, dexamethasone; FA, fluocinolone acetonide; IVTA, intravitreal triamcinolone acetonide; IVB, intravitreal bevacizumab; IVR, intravitreal ranibizumab; SOC, standard of care; IOP, intraocular pressure.

The risk of bias in the included RCTs was assessed by the Cochrane “risk of bias” tool according to seven standards (Barcot et al., 2019). The bias risk items are summarized in Supplementary Figure S4. Most of these RCTs were judged as low-bias-risk indicating good quality. Network meta-analysis diagrams of RCTs are provided in Figure 1.

### Outcomes

**FIGURE 2:**
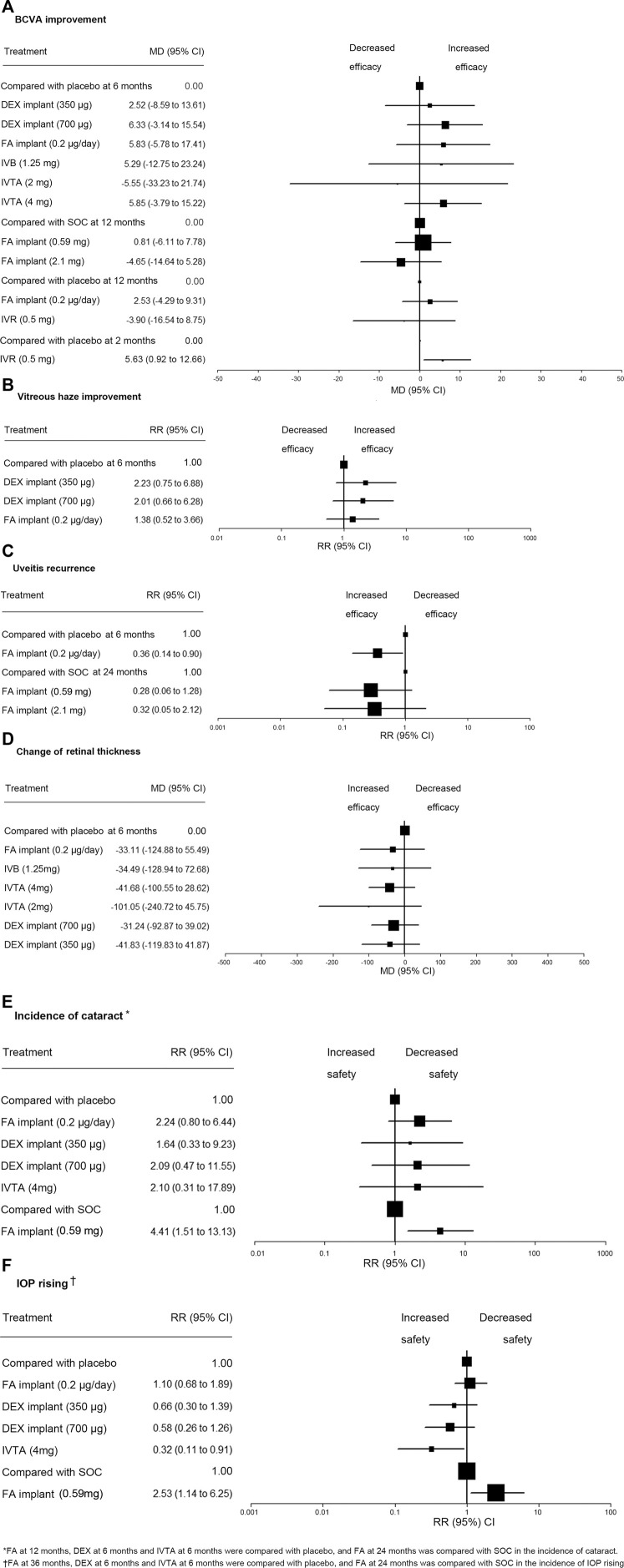
Network meta-analysis for all outcomes and ranking of the safety of IOP rising. **(A)** Comparisons of treatments for BCVA improvement. **(B)** Comparisons of treatments for vitreous haze improvement. **(C)** Comparisons of treatments for uveitis recurrence. **(D)** Comparisons of treatments for change of retinal thickness. **(E)** Comparisons of treatments for incidence of cataract. **(F)** Comparisons of treatments for IOP rising. RR, risk ratio; MD, mean deviations; BCVA, best-corrected visual acuity; DEX, dexamethasone; FA, fluocinolone acetonide; IVTA, intravitreal triamcinolone acetonide; IVB, intravitreal bevacizumab; IVR, intravitreal ranibizumab; SOC, standard of care; IOP, intraocular pressure.

Using the data from the studies mentioned, we assessed BCVA change from a baseline of 11 treatments (Lowder et al., 1960; Soheilian et al., 2010; Rahimi et al., 2012; Shin and Yu, 2015; Staurenghi et al., 2018; Lai et al., 2018; Thorne et al., 2019; Jaffe et al., 2020) (Figure 2A), percentage of eyes achieving a vitreous haze grading of zero of four treatments (Lowder et al., 1960; Jaffe et al., 2020) (Figure 2B), uveitis recurrence rate of five treatments (Pavesio et al., 2010; Sangwan et al., 2015; Jaffe et al., 2020) (Figure 2C), and retinal thickness change from baseline of seven treatments (Lowder et al., 1960; Soheilian et al., 2010; Rahimi et al., 2012; Shin and Yu, 2015; Thorne et al., 2019; Jaffe et al., 2020) (Figure 2D) in network meta-analysis. We compared incidence of cataract of seven treatments (Lowder et al., 1960; EMC, 2012; Shin and Yu, 2015; Thorne et al., 2019; Jaffe et al., 2020) (Figure 2E) and use of IOP-lowering medications after a baseline of seven treatments (Lowder et al., 1960; Pavesio et al., 2010; EMC, 2012; Jaffe et al., 2020) (Figure 2F) in network meta-analysis.

### BCVA Improvement

In pairwise comparison, a significant difference (*p* < .05) was detected in these five comparisons. DEX implant, 700 µg (MD 7.10, 95% CI 1.68–12.52), FA implant, 0.2 µg/day (MD 5.85, 95% CI 0.94–10.76), and IVTA, 4 mg (MD 4.85, 95% CI (3.47–6.23) were associated with greater improvement from baseline BCVA compared with placebo at 6 months, and FA implant, 0.59 mg, was associated with greater improvement from baseline BCVA compared to FA implant, 2.1 mg (MD 5.50, 95% CI 3.44–7.56), and SOC (MD 0.82, 95% CI 0.55–1.09) at 12 months. Compared with placebo, IVR was associated with a significantly higher improvement of BCVA at 2 months (MD 5.68, 95% CI 1.00–10.36) (Table 1).

**TABLE 1:** Pairwise comparisons of efficacy in RCTs.

Treatment 1	Treatment 2	Pairwise meta-analysis					Network meta-analysis		*p* value for test of inconsistency^d^
		No. of comparisons	RR (95%CI)^a^	MD (95%CI)^b^	I-squared (%)^c^	Favors	Pooled RR (95% CI)^a^	Pooled MD (95% CI)^b^	
**BCVA change from baseline (at 6 months)**									
DEX implant (700 µg)	DEX implant (350 µg)	1	NA	4.20 (−1.71–10.11)	NA	DEX implant (700 µg)	NA	3.83 (−7.37–14.71)	NA
DEX implant (700 µg)	FA implant (0.2 µg/day)	0	NA	NA	NA	NA	NA	0.49 (−14.48–15.40)	NA
DEX implant (700 µg)	IVB (1.25 mg)	0	NA	NA	NA	NA	NA	0.96 (−17.14–18.83)	NA
DEX implant (700 µg)	IVTA (2 mg)	0	NA	NA	NA	NA	NA	11.79 (−15.50–39.57)	NA
DEX implant (700 µg)	Placebo	1	NA	7.10 (1.68–12.52)	NA	DEX implant (700 µg)	NA	6.33 (−3.14–15.54)	0.72
DEX implant (350 µg)	FA implant (0.2 µg/day)	0	NA	NA	NA	NA	NA	−3.31 (−19.29 to 12.85)	NA
DEX implant (350 µg)	IVB (1.25 mg)	0	NA	NA	NA	NA	NA	−2.82 (−22.73 to 17.00)	NA
DEX implant (350 µg)	IVTA (2 mg)	0	NA	NA	NA	NA	NA	8.04 (−20.12–36.83)	NA
DEX implant (350 µg)	IVTA (4 mg)	0	NA	NA	NA	NA	NA	−3.35 (−16.22 to 9.62)	NA
DEX implant (350 µg)	Placebo	1	NA	2.90 (−1.46–7.26)	NA	DEX implant (350 µg)	NA	2.52 (−8.59–13.61)	NA
FA implant (0.2 µg/day)	IVB (1.25 mg)	0	NA	NA	NA	NA	NA	0.51 (−17.14–18.83)	NA
FA implant (0.2 µg/day)	IVTA (2 mg)	0	NA	NA	NA	NA	NA	11.37 (−18.22–41.04)	NA
FA implant (0.2 µg/day)	IVTA (4 mg)	0	NA	NA	NA	NA	NA	−0.03 (−15.17 to 14.89)	NA
FA implant (0.2 µg/day)	Placebo	1	NA	5.85 (0.94–10.76)	NA	FA implant (0.2 µg/day)	NA	5.83 (−5.78–17.41)	NA
IVB (1.25 mg)	IVTA (2 mg)	1	NA	11.00 (−6.76–28.76)	NA	IVB (1.25 mg)	NA	10.71 (−9.54–31.76)	NA
IVTA (2 mg)	IVTA (4 mg)	0	NA	NA	NA	NA	NA	−11.35 (−37.54 to 14.33)	NA
IVTA (4 mg)	DEX implant (700 µg)	1	NA	0.39 (−0.44–1.22)	NA	IVTA (4 mg)	NA	−0.46 (−10.00 to 9.02)	0.72
IVTA (4 mg)	IVB (1.25 mg)	1	NA	0.50 (−10.83–11.83)	NA	IVTA (4 mg)	NA	0.52 (−15.02–16.06)	NA
IVTA (4 mg)	Placebo	1	NA	4.85 (3.47–6.23)	NA	IVTA (4 mg)	NA	5.85 (−3.79–15.22)	0.73
**BCVA change from baseline (at 12 months)**									
FA implant (0.59 mg)	FA implant (2.1 mg)	1	NA	5.50 (3.44–7.56)	NA	FA implant (0.59 mg)	NA	5.46 (−1.67–12.59)	NA
FA implant (0.59 mg)	SOC	1	NA	0.82 (0.55–1.09)	NA	FA implant (0.59 mg)	NA	0.81 (−6.11–7.78)	NA
FA implant (2.1 mg)	SOC	0	NA	NA	NA	NA	NA	−4.65 (−14.64 to 5.28)	NA
FA implant (0.2 µg/day)	Placebo	1	NA	2.50 (−2.40–7.40)	NA	FA implant (0.2 µg/day)	NA	2.53 (−4.29–9.31)	NA
IVR (0.5 mg)	Placebo	1	NA	4.10 (−8.04–16.24)	NA	Placebo	NA	3.90 (−7.90–20.87)	NA
FA implant (0.2 µg/day)	IVR (0.5 mg)	0	NA	NA	NA	NA	NA	6.39 (−7.90–20.87)	NA
**BCVA change from baseline (at 2 months)**									
IVR (0.5 mg)	Placebo	2	NA	5.68 (1.00–10.36)	0.0	IVR (0.5 mg)	NA	5.63 (0.92–12.66)^e^	NA
**Achieving a vitreous haze grading of 0 (at 6 months)**									
DEX implant (350 µg)	DEX implant (700 µg)	1	1.11 (0.68–1.79)	NA	NA	DEX implant (350 µg)	1.10 (0.39–3.10)	NA	NA
DEX implant (350 µg)	FA implant (0.2 µg/day)	0	NA	NA	NA	NA	1.62 (0.37–7.16)	NA	NA
DEX implant (350 µg)	Placebo	1	2.18 (1.15–4.13)	NA	NA	DEX implant (350 µg)	2.23 (0.75–6.88)	NA	NA
DEX implant (700 µg)	FA implant (0.2 µg/day)	0	NA	NA	NA	NA	1.46 (0.33–6.53)	NA	NA
DEX implant (700 µg)	Placebo	1	1.97 (1.03–3.78)	NA	NA	DEX implant (700 µg)	2.01 (0.66–6.28)	NA	NA
FA implant (0.2 µg/day)	Placebo	1	1.35 (1.03–1.77)	NA	NA	FA implant (0.2 µg/day)	1.38 (0.52–3.66)	NA	NA
**Uveitis recurrence (at 6 months)**									
FA implant (0.2 µg/day)	Placebo	2	0.36 (0.25–0.50)	NA	87.7	FA implant (0.2 µg/day)	0.36 (0.14–0.90)^e^	NA	NA
**Uveitis recurrence (at 24 months)**									
2FA implant (0.59 mg)	FA implant (2.1 mg)	2	0.89 (0.44–1.79)	NA	67.2	FA implant (0.59 mg)	0.88 (0.29–2.56)	NA	NA
FA implant (0.59 mg)	SOC	1	0.29 (0.17–0.49)	NA	NA	FA implant (0.59 mg)	0.28 (0.06–1.28)	NA	NA
FA implant (2.1 mg)	SOC	0	NA	NA	NA	NA	0.32 (0.05–2.12)	NA	NA
**Retinal thickness change from baseline (at 6 months)**									
DEX implant (350 µg)	DEX implant (700 µg)	1	NA	−17.90 (−56.66 to 20.86)	NA	DEX implant (350 µg)	NA	−11.13 (−92.87 to 67.24)	NA
DEX implant (350 µg)	FA implant (0.2 µg/day)	0	NA	NA	NA	NA	NA	−7.20 (−126.6 to 115.7)	NA
DEX implant (350 µg)	IVB (1.25 mg)	0	NA	NA	NA	NA	NA	−8.58 (−129.2 to 107.5)	NA
DEX implant (350 µg)	IVTA (2 mg)	0	NA	NA	NA	NA	NA	60.37 (−98.80–215.0)	NA
DEX implant (350 µg)	IVTA (4 mg)	0	NA	NA	NA	NA	NA	−1.33 (−93.90 to 88.18)	NA
DEX implant (350 µg)	Placebo	1	NA	−32.60 (−76.12 to 10.92)	NA	DEX implant (350 µg)	NA	−41.83 (−119.83 to 41.87)	NA
DEX implant (700 µg)	FA implant (0.2 µg/day)	0	NA	NA	NA	NA	NA	4.00 (−102.8–119.2)	NA
DEX implant (700 µg)	IVB (1.25 mg)	0	NA	NA	NA	NA	NA	2.68 (−98.60–101.3)	NA
DEX implant (700 µg)	IVTA (2 mg)	0	NA	NA	NA	NA	NA	71.72 (−72.09–214.97)	NA
DEX implant (700 µg)	IVTA (4 mg)	1	NA	−0.51 (−26.95 to 25.93)	NA	DEX implant (700 µg)	NA	10.28 (−55.30–73.51)	0.57
DEX implant (700 µg)	Placebo	1	NA	−14.70 (−52.75 to 23.35)	NA	DEX implant (700 µg)	NA	−31.24 (−92.87 to 39.02)	0.57
FA implant (0.2 µg/day)	IVB (1.25 mg)	0	NA	NA	NA	NA	NA	−1.37 (−140.58 to 126.97)	NA
FA implant (0.2 µg/day)	IVTA (2 mg)	0	NA	NA	NA	NA	NA	66.91 (-106.56–232.33)	NA
FA implant (0.2 µg/day)	IVTA (4 mg)	0	NA	NA	NA	NA	NA	6.06 (−109.05–111.63)	NA
FA implant (0.2 µg/day)	Placebo	1	NA	−34.00 (−88.55 to 20.55)	NA	FA implant (0.2 µg/day)	NA	−33.11 (−124.88 to 55.49)	NA
IVTA (2 mg)	IVTA (4 mg)	0	NA	NA	NA	NA	NA	−61.43 (−189.87 to 67.19)	NA
IVTA (4 mg)	Placebo	1	NA	−46.30 (−52.64 to −39.66)	NA	IVTA (4 mg)	NA	−41.68 (−100.55 to 28.62)	0.55
IVTA (4 mg)	IVB (1.25 mg)	1	NA	−7.54 (−12.54 to −2.54)	NA	IVTA (4 mg)	NA	−7.52 (−85.16 to 67.79)	NA
IVTA (2 mg)	IVB (1.25 mg)	1	NA	−69.40 (−149.14 to 10.34)	NA	IVTA (2 mg)	NA	−69.17 (−175.36 to 36.67)	NA

Abbreviations: RR, risk ratios; MD, mean deviations; BCVA, best-corrected visual acuity; DEX, dexamethasone; FA, fluocinolone acetonide; IVTA, intravitreal triamcinolone acetonide; IVB, intravitreal bevacizumab; IVR, intravitreal ranibizumab; SOC, standard of care; IOP, intraocular pressure; NA, not available.

a The risk ratios (95% CI) were the result of comparing the treatment 1 regimens with the treatment 2 regimens (the reference group).

b The mean deviations (95% CI) were the result of comparing the treatment 1 regimens with the treatment 2 regimens (the reference group).

c Heterogeneity was assessed by the I^2^ test, with an I^2^ > 50% considered as the existence of significant heterogeneity.

d The results of the test for inconsistency were incorporated; *p* < .05 indicates existence of inconsistency.

e Statistically significant (*p* < .05).

In Bayesian network meta-analysis, there was no significant difference in efficacy of improving BCVA among those treatments (Table 1). We compared BCVA change from baseline at 6 months of six treatment regimens with that of the placebo, and no significant difference was detected. Two FA implants revealed no significant difference compared with SOC in BCVA change from baseline at 12 months. FA implant, 0.2 µg/day, and IVR, 0.5 mg, also showed no significant difference compared with placebo in BCVA change from baseline at 12 months. Compared with placebo, IVR was associated with a significant efficacy of improving BCVA at 2 months (MD 5.63, 95% CI 0.92–12.66) (Figure 2A).

The ranking probabilities of an intravitreal therapeutic agent at any possible position are presented in Table 3. The probabilities, whereby FA implant, 0.2 µg/day, ranked as the first, second, third, fourth, fifth, sixth, and seventh most efficacious drug for improving BCVA at 6 months was 31%, 17%, 15%, 14%, 12%, 7%, and 4%. The rank of interventions improving BCVA at 12 months was presented in Table 3.

### Vitreous Haze Improvement

In pairwise meta-analysis, DEX implant, 350 µg (RR 2.18, 95% CI 0.68–1.79), DEX implant, 700 µg (RR 1.97, 95% CI 1.03–3.78), FA implant, 0.2 µg/day (RR 1.35, 95% CI 1.03–1.77) at 6 months were associated with an increased rate of achieving a vitreous haze grading of zero compared with placebo with statistical significance (*p* < .05) (Table 1). In Bayesian network meta-analysis (Table 1), an identical trend was detected, but the difference was not statistically significant among drugs. We compared the efficacy of three intravitreal implants with that of the placebo, and there was no significant difference among drugs in RCTs (Figure 2B). The probabilities of DEX implant, 350 µg, ranked as the first, second, third, or fourth effective treatment for improving vitreous haze at 6 months was 51%, 34%, 11%, and 4%, respectively (Table 3).

### Uveitis Recurrence

In pairwise meta-analysis, patients in the FA implant, 0.2 µg/day, group were associated with a lower risk of uveitis recurrence than those in the placebo group at 6 months (RR 0.36, 95% CI 0.25 to 0.50, *p* < .05), and FA implant, 0.59 mg, was associated with lower risk of recurrence than the SOC group at 24 months (RR 0.29, 95% CI 0.17 to 0.49, *p* < .05) (Table 1). Considerable heterogeneity was detected in the comparison between FA implant, 0.2 µg/day, and placebo (I-squared = 87.7%) or between FA, 0.59 mg, and FA, 2.1 mg (I-squared = 67.2%).

In Bayesian network meta-analysis, there was no significant difference in uveitis recurrence at 24 months among drugs in RCTs. Uveitis recurrence rate at 6 months of FA implant, 0.2 µg/day, was significantly lower than that of placebo (RR 0.36, 95% CI 0.14 to 0.90, *p* < .05) (Figure 2C). The chances of the FA implant, 0.59 mg, being ranked as the first, second, and third most effective clinical intervention for reducing the relapse of noninfectious uveitis at 24 months was 60%, 38%, and 2% (Table 3).

### Change of Retinal Thickness

In pairwise meta-analysis, a statistically significant difference in the change of retinal thickness was found when comparing IVTA, 4 mg, versus placebo (MD −46.30, 95% CI −52.64 to −39.66, *p* < .05) and IVTA, 4 mg, versus IVB, 1.25 mg (MD −7.54, 95% CI −12.54 to −2.54, *p* < .05) at 6 months (Table 1). Bayesian network meta-analysis showed no significant difference in the change of retinal thickness among seven treatments at 6 months (Table 1). We compared the efficacy of six intravitreal therapeutics agents with that of placebo, and no significant difference was observed (Figure 2D). The probability of IVTA, 2 mg, ranking as the first to seventh best intervention for lowest retinal thickness at 6 months were 70%, 11%, 5%, 4%, 3%, 3%, and 1% (Table 3).

### Incidence of Cataract

In pairwise comparison, there were statistically significant differences when comparing the incidence of cataract in FA implant, 0.59 mg, versus SOC (RR 4.33, 95% CI 2.97 to 6.33, *p* < .05) at 24 months or FA implant, 0.2 µg/day, versus placebo (RR 2.15, 95% CI 1.08 to 4.25, *p* < .05) at 12 months (Table 2), and no heterogeneity was detected in both comparisons (I-squared = 0.0%).

**TABLE 2:** Pairwise comparisons of safety in RCTs.

Treatment 1	Treatment 2	Pairwise meta-analysis			Network meta-analysis		*p* value for test of inconsistency^c^
		No. of comparisons	RR (95% CI)^a^	I-squared (%)^b^	Favors	Pooled RR (95% CI)^a^	
**Incidence of cataract (FA 12 months vs. DEX 6 months vs. IVTA 6 months)**							
DEX implant (700 µg)	DEX implant (350 µg)	1	1.23 (0.47–3.24)	NA	DEX implant (350 µg)	1.26 (0.30–5.83)	NA
DEX implant (700 µg)	FA implant (0.2 µg/day)	0	NA	NA	NA	0.97 (0.16–7.04)	NA
DEX implant (700 µg)	Placebo	1	2.00 (0.65–6.12)	NA	Placebo	2.09 (0.47–11.55)	0.75
DEX implant (350 µg)	FA implant (0.2 µg/day)	0	NA	NA	NA	0.77 (0.11–5.73)	NA
DEX implant (350 µg)	IVTA (4 mg)	0	NA	NA	NA	0.81 (0.05–10.60)	NA
DEX implant (350 µg)	Placebo	1	1.62 (0.48–5.41)	NA	Placebo	1.64 (0.33–9.23)	NA
FA implant (0.2 µg/day)	IVTA (4 mg)	0	NA	NA	NA	1.08 (0.09–9.55)	NA
FA implant (0.2 µg/day)	Placebo	2	2.15 (1.08–4.25)	0.0	Placebo	2.24 (0.80–6.44)	NA
IVTA (4 mg)	DEX implant (700 µg)	1	0.01 (0.00–2.72 × 10^7^)	NA	IVTA (4 mg)	0.98 (0.07–13.97)	0.69
IVTA (4 mg)	Placebo	1	2.00 (0.40–9.95)	NA	Placebo	2.10 (0.31–17.89)	0.80
**Incidence of cataract (at 24 months)**							
FA implant (0.59 mg)	SOC	2	4.33 (2.97–6.33)	0.0	SOC	4.41 (1.51–13.13)^d^	NA
**Using of IOP-lowering medications (FA 36 months vs. DEX 6 months vs. IVTA 6 months)**							
DEX implant (700 µg)	DEX implant (350 µg)	1	0.88 (0.50–1.57)	NA	DEX implant (700 µg)	0.88 (0.38–2.04)	NA
DEX implant (700 µg)	FA implant (0.2 µg/day)	0	NA	NA	NA	0.52 (0.20–1.29)	NA
DEX implant (700 µg)	IVTA (4 mg)	1	1.80 (1.34–2.42)	NA	IVTA (4 mg)	1.80 (0.90–3.63)	NA
DEX implant (700 µg)	Placebo	1	0.58 (0.35–0.96)	NA	DEX implant (700 µg)	0.58 (0.26–1.26)	NA
DEX implant (350 µg)	FA implant (0.2 µg/day)	0	NA	NA	NA	0.60 (0.23–1.43)	NA
DEX implant (350 µg)	IVTA (4 mg)	0	NA	NA	NA	2.04 (0.70–6.04)	NA
DEX implant (350 µg)	Placebo	1	0.66 (0.40–1.06)	NA	DEX implant (350 µg)	0.66 (0.30–1.39)	NA
FA implant (0.2 µg/day)	IVTA (4 mg)	0	NA	NA	NA	3.43 (1.12–11.35)^d^	NA
FA implant (0.2 µg/day)	Placebo	2	1.05 (0.87–1.26)	0.0	Placebo	1.10 (0.68–1.89)	NA
Placebo	IVTA (4 mg)	0	NA	NA	NA	3.13 (1.10–9.00)^d^	NA
**Using of IOP-lowering medications (at 24 months)**							
FA implant (0.59 mg)	SOC	2	2.42 (1.94–3.01)	13.5	SOC	2.53 (1.14–6.25)^d^	NA

Abbreviations: RR, risk ratios; MD, mean deviations; BCVA, best-corrected visual acuity; DEX, dexamethasone; FA, fluocinolone acetonide; IVTA, intravitreal triamcinolone acetonide; IVB, intravitreal bevacizumab; SOC, standard of care; IOP, intraocular pressure; NA, not available.

a The odds ratios (95% CI) were the result of comparing the treatment 1 regimens with the treatment 2 regimens (the reference group).

b Heterogeneity was assessed by the I^2^ test, with an I^2^ > 50% considered as the existence of significant heterogeneity.

c The results of the test for inconsistency were incorporated; *p* < .05 indicates existence of inconsistency.

d Statistically significant.

In Bayesian network meta-analysis (Table 2), a statistically significant result in the incidence of cataract was detected between FA implant, 0.59 mg, and SOC at 24 months (RR 4.41, 95% CI (1.51–13.13, *p* < .05). We compared the incidence of cataract of four intravitreal therapeutics regimens, and no significant difference was detected. There were no significant differences in the treatments compared with placebo in the incidence of cataract (Figure 2E). The probabilities of DEX implant, 350 µg, ranked as the first to fifth intervention that associated with lowest risk of cataracts were 18%, 21%, 24%, 23%, and 14% (Table 3).

**TABLE 3:** Ranking probabilities of each intervention with different outcomes at any position.

Intervention	Rank 1	Rank 2	Rank 3	Rank 4	Rank 5	Rank 6	Rank 7
**BCVA improvement at 6 months**							
DEX implant (350 µg)	0.07	0.09	0.11	0.20	0.25	0.19	0.09
DEX implant (700 µg)	0.15	0.24	0.26	0.20	0.10	0.03	0.01
FA implant (0.2 µg/day)	0.31	0.17	0.15	0.14	0.12	0.07	0.04
IVB (1.25 mg)	0.28	0.15	0.10	0.12	0.12	0.20	0.03
IVTA (2 mg)	0.07	0.07	0.04	0.05	0.06	0.08	0.63
IVTA (4 mg)	0.11	0.27	0.30	0.19	0.09	0.03	0.01
Placebo	0.00	0.01	0.04	0.10	0.26	0.39	0.20
**BCVA improvement at 12 months**							
FA implant (0.59 mg)	0.64	0.34	0.02	NA	NA	NA	NA
FA implant (2.1 mg)	0.04	0.10	0.86	NA	NA	NA	NA
SOC	0.32	0.57	0.11	NA	NA	NA	NA
**BCVA improvement at 12 months**							
IVR (0.5 mg)	0.17	0.13	0.70	NA	NA	NA	NA
FA implant (0.2 µg/day)	0.67	0.24	0.09	NA	NA	NA	NA
Placebo	0.16	0.63	0.21	NA	NA	NA	NA
**Vitreous haze improvement at 6 months**							
DEX implant (350 µg)	0.51	0.34	0.11	0.04	NA	NA	NA
DEX implant (700 µg)	0.33	0.45	0.16	0.07	NA	NA	NA
FA implant (0.2 µg/day)	0.15	0.17	0.51	0.17	NA	NA	NA
Placebo	0.01	0.04	0.23	0.72	NA	NA	NA
**Uveitis recurrence at 24 months**							
FA implant (0.59 mg)	0.60	0.38	0.02	NA	NA	NA	NA
FA implant (2.1 mg)	0.36	0.56	0.08	NA	NA	NA	NA
SOC	0.03	0.06	0.90	NA	NA	NA	NA
**Change of retinal thickness at 6 months**							
DEX implant (350 µg)	0.11	0.25	0.17	0.14	0.15	0.12	0.07
DEX implant (700 µg)	0.03	0.10	0.17	0.21	0.25	0.18	0.06
FA implant (0.2 µg/day)	0.11	0.20	0.11	0.11	0.12	0.20	0.15
IVB (1.25 mg)	0.02	0.15	0.17	0.19	0.19	0.16	0.11
IVTA (2 mg)	0.70	0.11	0.05	0.04	0.03	0.03	0.03
IVTA (4 mg)	0.03	0.18	0.30	0.26	0.15	0.06	0.02
Placebo	0.00	0.01	0.03	0.05	0.11	0.25	0.55
**Incidence of cataract (FA 12 months vs. DEX 6 months vs. IVTA 6 months)**							
DEX implant (350 µg)	0.18	0.21	0.24	0.23	0.14	NA	NA
DEX implant (700 µg)	0.08	0.14	0.24	0.29	0.25	NA	NA
FA implant (0.2 µg/day)	0.03	0.17	0.24	0.28	0.28	NA	NA
IVTA (4 mg)	0.18	0.16	0.15	0.18	0.33	NA	NA
Placebo	0.52	0.33	0.13	0.03	0.00	NA	NA
**IOP rising (FA 36 months vs. DEX 6 months vs. IVTA 6 months)**							
DEX implant (350 µg)	0.07	0.29	0.51	0.07	0.06	NA	NA
DEX implant (700 µg)	0.02	0.59	0.31	0.04	0.03	NA	NA
FA implant (0.2 µg/day)	0.01	0.02	0.07	0.25	0.65	NA	NA
IVTA (4 mg)	0.90	0.07	0.02	0.01	0.01	NA	NA
Placebo	0.00	0.02	0.09	0.63	0.25	NA	NA

Abbreviations: BCVA, best-corrected visual acuity; DEX, dexamethasone; FA, fluocinolone acetonide; IVTA, intravitreal triamcinolone acetonide; IVB, intravitreal bevacizumab; IVR, intravitreal ranibizumab; SOC, standard of care; IOP, intraocular pressure; NA, not available.

### Intraocular Pressure

In pairwise comparison (Table 2), patients in the FA implant, 0.59 mg, group were associated with increased risk of using IOP-lowering medications at 24 months than those treated with SOC (RR 2.42, 95% CI 1.94 to 3.01, *p* < .05). Heterogeneity was within acceptable limits (I-squared = 13.5%). Comparison of IOP rising at 6 months between DEX implant, 700 µg, and IVTA, 4 mg (RR 1.80 95% CI 1.34 to 2.42, *p* < .05) showed a statistically significant difference.

In Bayesian network meta-analysis (Table 2), IVTA, 4 mg, at 6 months is shown to be associated with a lower risk of a high intraocular pressure compared with FA implant, 0.2 µg/day, at 36 months (RR 3.43 95% CI 1.12 to 11.35, *p* < .05). We compared the IOP rising of four intravitreal therapeutic agents with that of placebo, and IVTA, 4 mg, used significantly less IOP-lowering medications than that of placebo (RR 0.32 95% CI 0.11 to 0.91, *p* < .05) (Figure 2F). FA implant, 0.59 mg, caused significantly more IOP rising than SOC at 24 months (RR 2.53 95% CI 1.14 to 6.25, *p* < .05) (Figure 2F). The probabilities of IVTA, 4 mg, ranking as the first to fourth intervention showing the lowest incidence of an elevated IOP was: 90, 7, 7, 2, 1, and 1% (Table 3).

### Inconsistency and Ranking

The node-splitting approach (Higgins et al., 2012; Dias et al., 2013) was used to assess inconsistency and demonstrates that all *p*-values were higher than .05 and varied from .55 to .80 (Tables 1, 2). Therefore, no significant inconsistency was detected, and the test of inconsistency was able to apply when direct head-to-head evidence was available.

Ranking depended on point estimates in pairwise comparison between an intravitreal therapeutics agent and placebo or SOC (Figure 2). (Rücker and Schwarzer, 2015) We created a ranking on the safety of IOP rising in RCTs. IVTA, 4 mg, might be the best intravitreal therapeutic regimen associated with low risk of IOP rising (RR 3.13 95% CI 1.10–9.00) (Figure 2F). Additionally, because there were no significant difference observed in other efficacy and safety outcomes of drugs, we were unable to obtain a ranking of treatment based on data currently available.

### Sensitivity Analysis

Sensitivity analysis was performed by removing studies that showed high heterogeneity in the pairwise meta-analysis (Tables 1, 2). For uveitis recurrence at 24 months, high heterogeneity was found in the comparison between FA implant, 2.1 mg, and FA implant, 0.59 mg. After conducting the Bayesian network meta-analysis in the remaining studies, no significant change was revealed in network meta-analysis, indicating the reliability of our study. High heterogeneity was also detected in comparison between FA, 0.2 µg/day, and placebo for uveitis recurrence at 6 months. However, all included studies provide overwhelming evidence that FA, 0.2 µg/day, was associated with a lower recurrence rate. Thus, omission of any included study would not alter the result, indicating the robustness of the statistical analysis.

## Discussion

### Summary of Evidence

To ensure that the indirect comparisons done in this study were based on the inclusion of comparable patient populations and using similar designs, we limited our review to a set of homogeneous trials with rigorous criteria, including DEX implant, 350 μg; DEX implant, 700 μg; FA implant, 0.2 µg/day; FA implant, 0.59 mg; FA implant, 2.1 mg; IVB, 1.25 mg; IVR, 0.5 mg and IVTA, 2 mg; and IVTA, 4 mg; which are novel intravitreal therapeutic agents for the treatment of noninfectious uveitis. This network meta-analysis focused on nine intravitreal therapeutic regimens and systemic therapy involving 1806 patients by deriving data from 13 RCTs. Overall, no specific treatment regimen showed a statistically significant advantage or disadvantage over another regimen with regard to efficacy of BCVA improvement, vitreous haze improvement, uveitis recurrence, and change of retinal thickness. In our study, we assessed the safety profile of the treatments by evaluating cataract occurrence and drug-induced IOP rise. FA implant, 0.59 mg, caused more side effects than SOC in general (Figures 2E,F). IVTA, 4 mg, is shown to cause less elevated IOP than other intravitreal therapeutic agents (Figure 2F).

### Comparison of FA Implants With Standard of Therapy

In the present network meta-analysis, FA implants tend to be more effective in long-term, targeted control of inflammation as compared with SOC, and the difference is close to significance, which caused fewer side effects of cataracts and elevated IOP (Figures 2C,E,F). However, during the long-term use of systemic immunosuppressive drugs, the side effects were not limited to the eye, which typically included hepatotoxicity, renal impairment, severe gastrointestinal upset, and nephrotoxicity (Jabs et al., 2000; McCluskey et al., 2000). In the management of chronic noninfectious uveitis, intravitreal steroid implants are a steroid-sparing agent to control inflammation and are able to reduce serious and intolerable side effects of SOC.

### Comparison With Other Reviews

As far as we know, this is the first comparison of the major different intravitreal therapeutic regimens for noninfectious uveitis using a Bayesian network meta-analysis. Lacking multiarm trials and difficult-to-conduct, high-quality, head-to-head RCTs especially among these newly developed intravitreal therapeutic agents made it necessary to apply a network meta-analysis. A Cochrane review (Brady et al., 2016) compared a fluocinolone acetonide implant with SOC and included two trials (625 eyes), whereas our study summarizes 13 studies with a total of 1806 eyes included. Unlike past studies that focus on comparing intravitreal implants for noninfectious uveitis only (Brady et al., 2016; Vieira et al., 2020), the present study thoroughly ranks intravitreal therapeutic regimens, including IVTA and intravitreal anti-VEGF. This distinction is important because 2 or 4 mg of TA is one of the most widely used intravitreal steroids in the treatment of noninfectious uveitis (Ganapathy et al., 2018), and anti-VEGF agents as nonsteroid intravitreal therapeutics are currently undergoing evaluation for efficacy of controlling uveitic complications (Thomas and Lin, 2020), whereas anti-VEGF agents were not approved by the FDA as an intravitreal treatment option for noninfectious uveitis yet.

## Limitations

There are limitations in this Bayesian network meta-analysis. First, although we carried out a thorough search in several major databases, the number of RCTs is still limited, which led to wide 95% CIs. Some of the evidence is based on a single comparison due to limited studies. In addition, due to lacking direct head-to-head RCTs, we are not able to check consistency between direct and indirect comparison using the node-splitting method. In this study, we include the results of all available RCTs that evaluated intravitreal therapeutic regimens in patients with noninfectious uveitis during the study period. Second, different maximum follow-up time of included studies might affect the outcomes. Further statistical analysis (e.g., meta-regression or subgroup analysis or stratification) should be done to reduce bias when more studies are available. Third, our results are influenced by the following factors: age, race, sample size of study, duration and severity of disease, active or quiescence of inflammation, and surgical skill of surgeons. However, given the lack of standardized protocol for studies investigating intravitreal therapeutic agents, heterogeneity was unavoidable. In the sensitivity analysis, our main results did not change after removing some studies, leading to high heterogeneity that proves the stability and reliability of our model. Fourth, given the lack of head-to-head RCTs, most of our evidence is derived from indirect comparisons, which might be a source of bias. However, in the absence of direct evidence, network meta-analysis of different treatment regimens may be valuable for clinical decision making. Fifth, we were not able to conduct subgroup analysis according to an anatomical classification due to the limited studies. It is important to establish the assumption that intermediate uveitis, posterior uveitis, or panuveitis respond to the analyzed interventions similarly. To the best of our knowledge, these three anatomical categories of uveitis usually share similar treatment strategies (systemic therapy and regional therapies, such as corticosteroid implants) and are commonly classified as a whole group in clinical trials. In addition, as described in previous meta-analysis (Brady et al., 2016; Vieira et al., 2020), it was also used as a population to evaluate the efficacy and safety of corticosteroid implants or immunomodulatory drugs. Additionally, the number of the RCTs included in this study is relatively small. It is not appropriate to perform subgroup analysis. Therefore, the subtype of uveitis may not be a factor resulting in heterogeneity, and the network meta-analysis was conducted properly. Sixth, different follow-up time points for each safety outcome might confound findings. In this study, we evaluated the drug efficacy at the same follow-up periods due to the data concerning the results of different interventions being available. For the evaluation of adverse effects, we were unable to assess the data at the same follow-up periods for all compared therapeutic regimens. For example, when we compared the incidence rate of cataract at 6 months among placebo, FA implants, DEX implants, and IVTA (Figure 2E), data from the FA implants at 6 months was not available. To reduce the bias, we used the data of FA implants at 12 months instead, which is the data closest to the 6-month follow-up time point.

## Conclusion

The present network meta-analysis results suggest that no intravitreal therapeutic regimens reported in this study show a significant advantage or disadvantage to another regimen with regard to efficacy. However, SOC, which is based on the use of systemic drugs, is associated with lower risk in view of the ocular side effects compared with FA implants. IVTA, 4 mg, might be a better choice than the other intravitreal therapeutic regimens for a lower risk of IOP rising. In the absence of evidence from head-to-head RCTs, network meta-analysis of different treatment regimens may be valuable for clinical decision making.

## Data Availability

The original contributions presented in the study are included in the article/Supplementary Material, further inquiries can be directed to the corresponding author.
