# Supplementary figures and images for: Comparative Efficacy and Safety of Advanced Intravitreal Therapeutic Agents for Noninfectious Uveitis: A Systematic Review and Network Meta-Analysis
Source: Front Pharmacol. 2022 Apr 5;13:749312. doi: 10.3389/fphar.2022.749312 (PMC9017745; doi:10.3389/fphar.2022.749312)

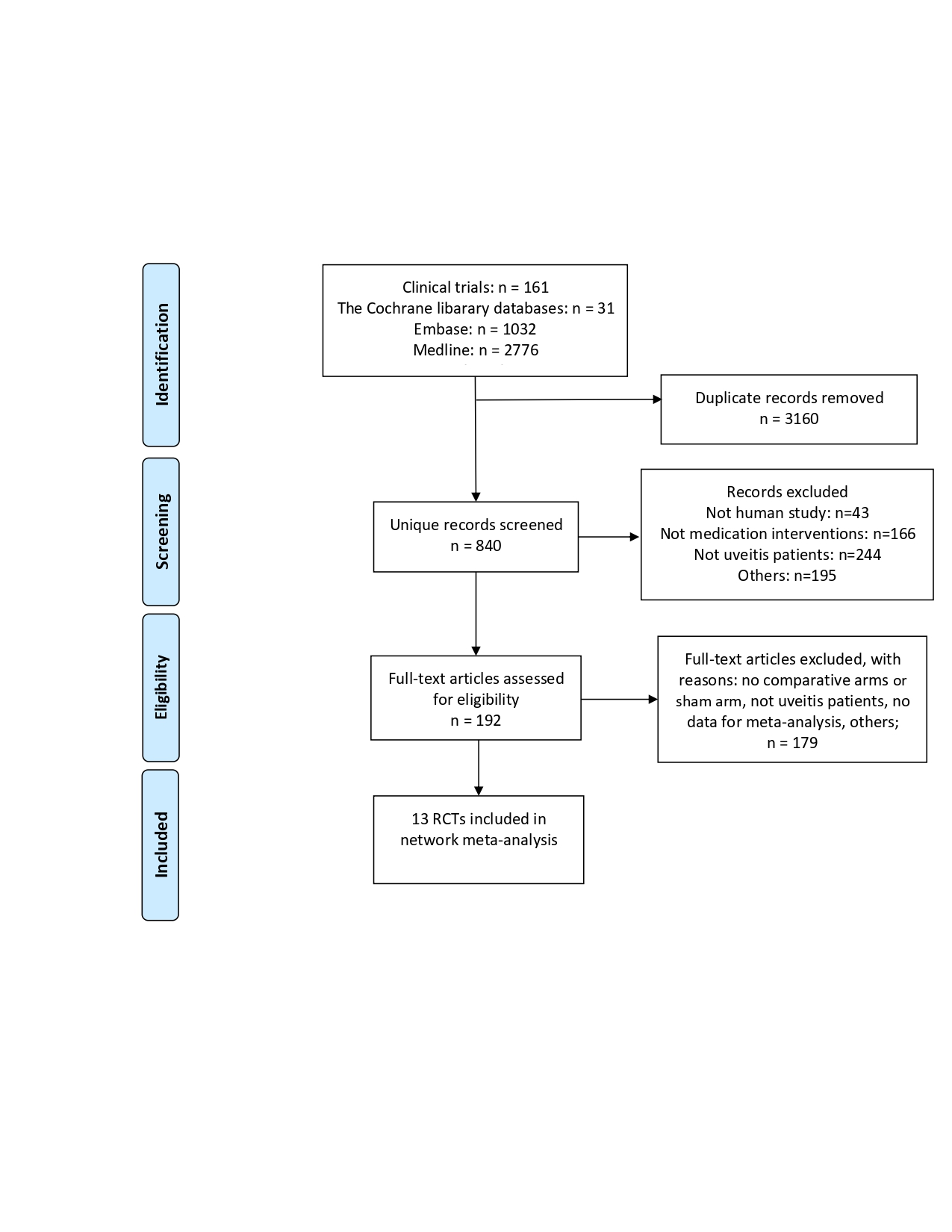

Supplement: Supplementary file 1 [file Image3.TIF]

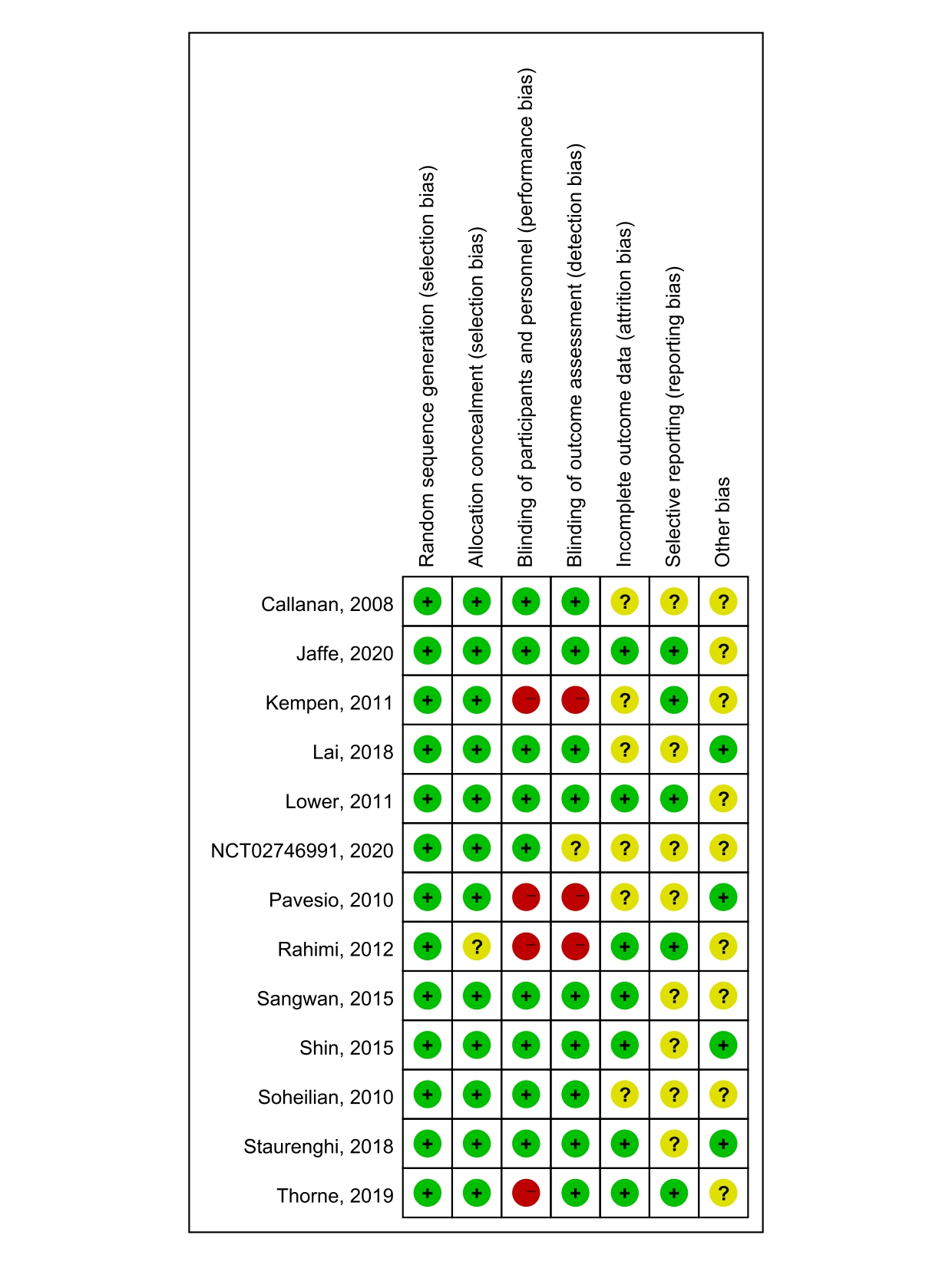

Supplement: Supplementary file 2 [file Image4.TIF]
