# Supplementary material for: Comparative Efficacy and Safety of Advanced Intravitreal Therapeutic Agents for Noninfectious Uveitis: A Systematic Review and Network Meta-Analysis
Source: Front Pharmacol. 2022 Apr 5;13:749312. doi: 10.3389/fphar.2022.749312 (PMC9017745; doi:10.3389/fphar.2022.749312)
Supplement: Supplementary file 3 [file Image2.PDF]

Supplementary Figure S2. Trace and density plot

Trace and density plots for all model parameters shows that Markov Monte Carlo chains converged well in general.

A. BCVA improvement

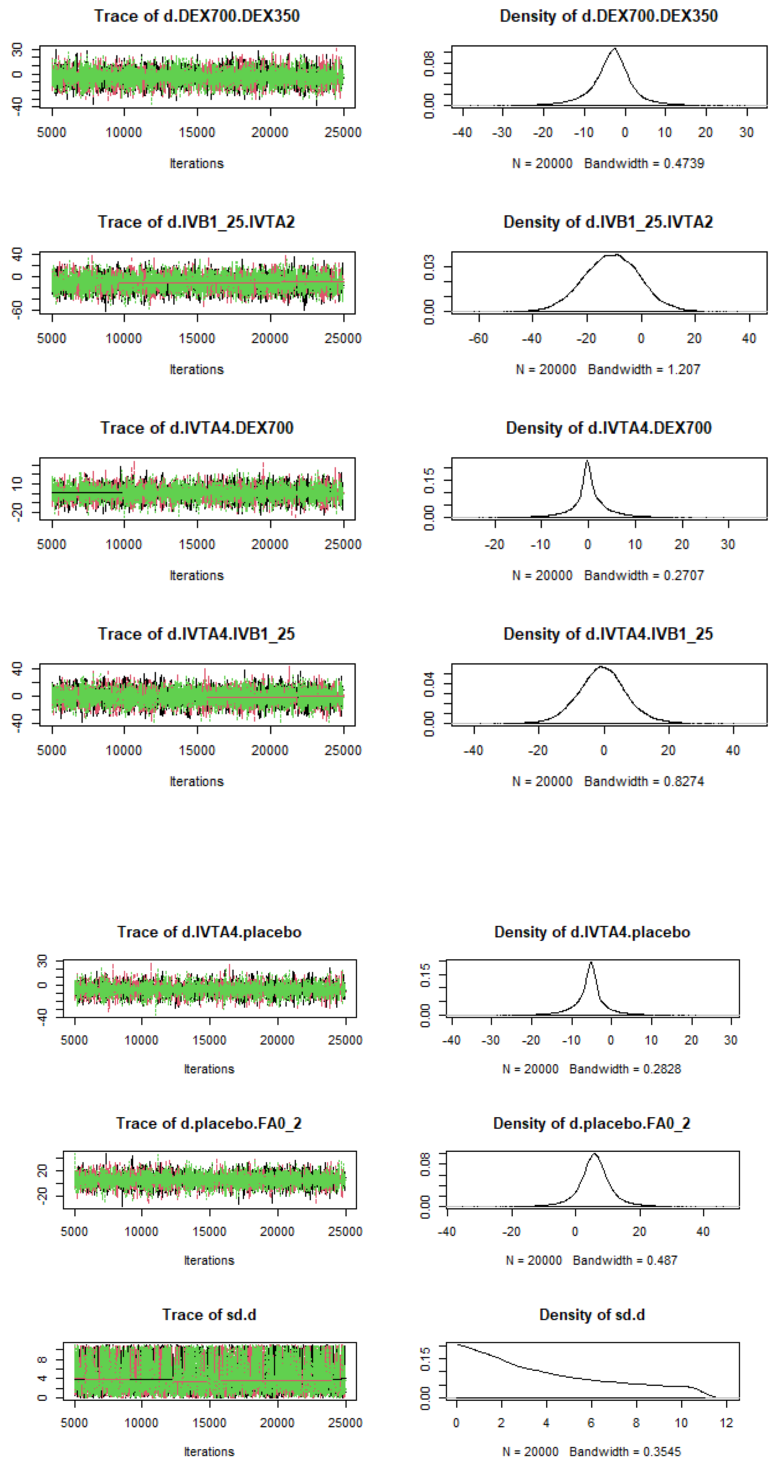

B. Vitreous haze improvement

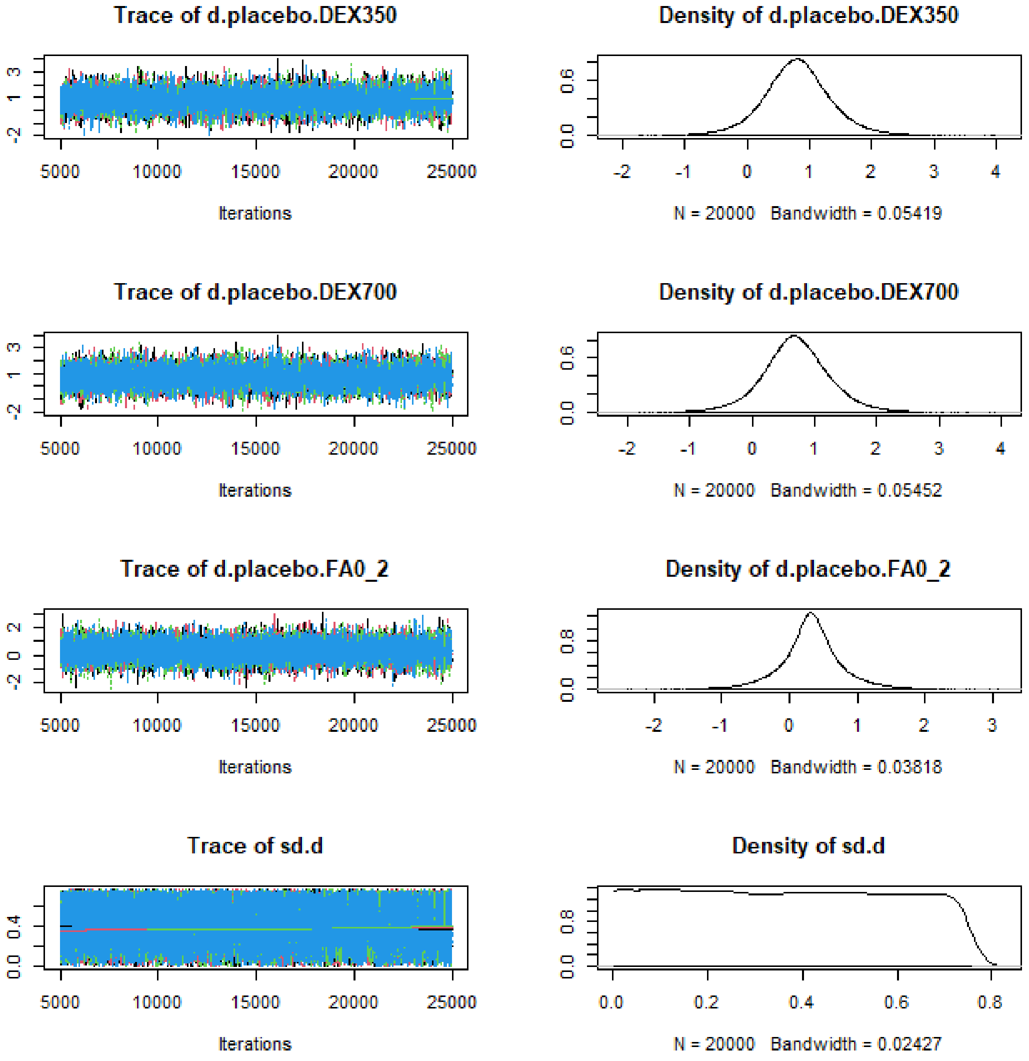

C. Uveitis recurrence

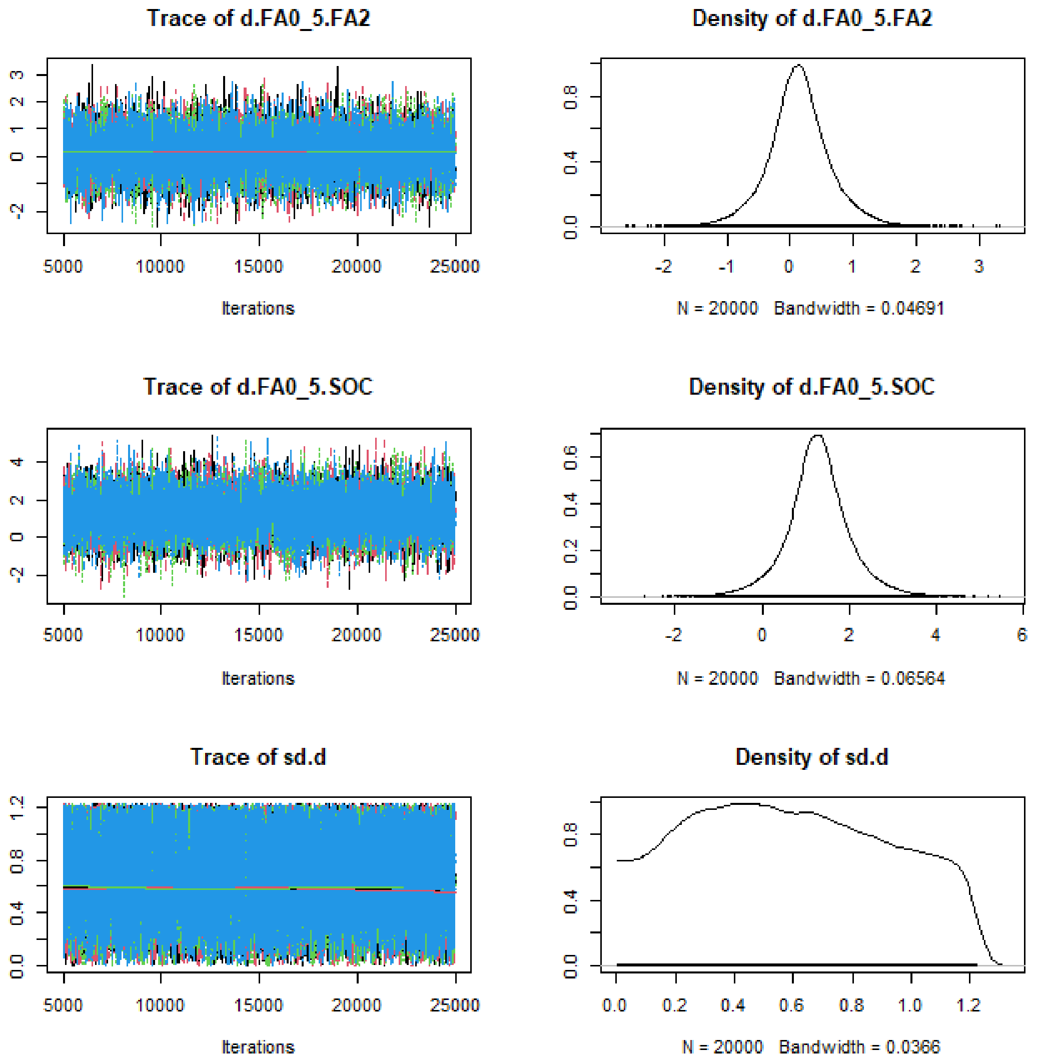

D. Change of macular retinal thickness

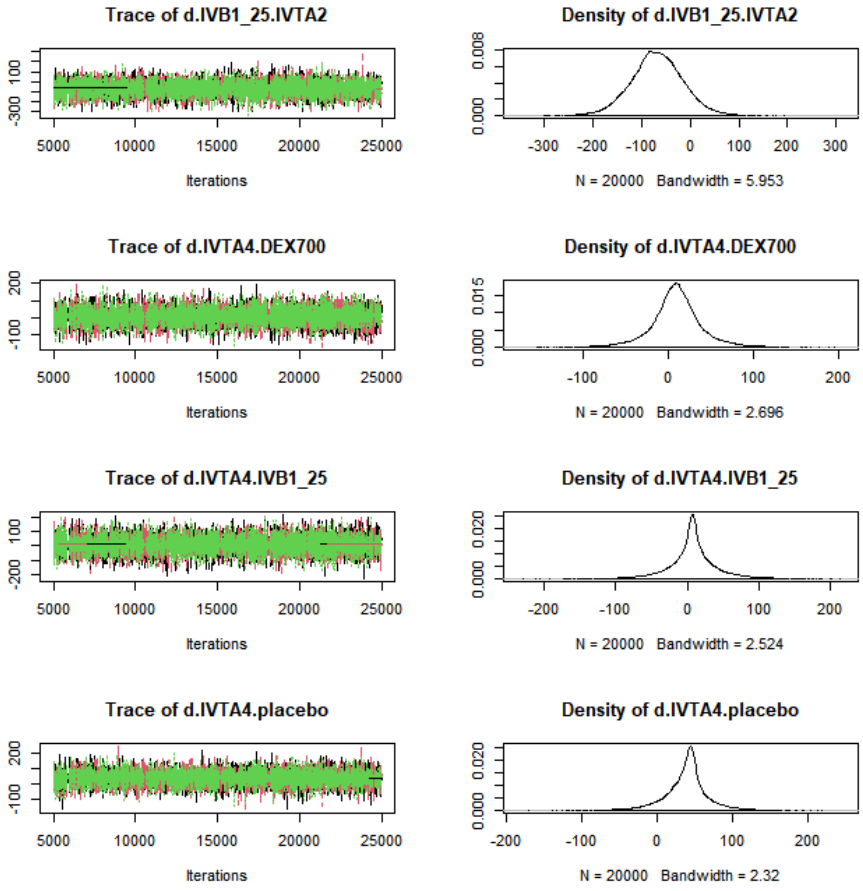

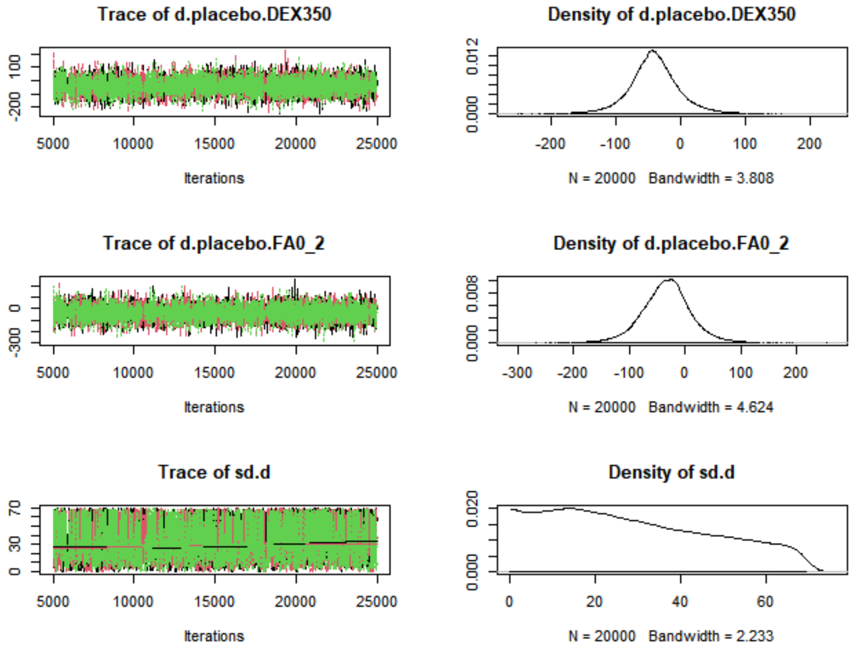

E. Incidence of cataract

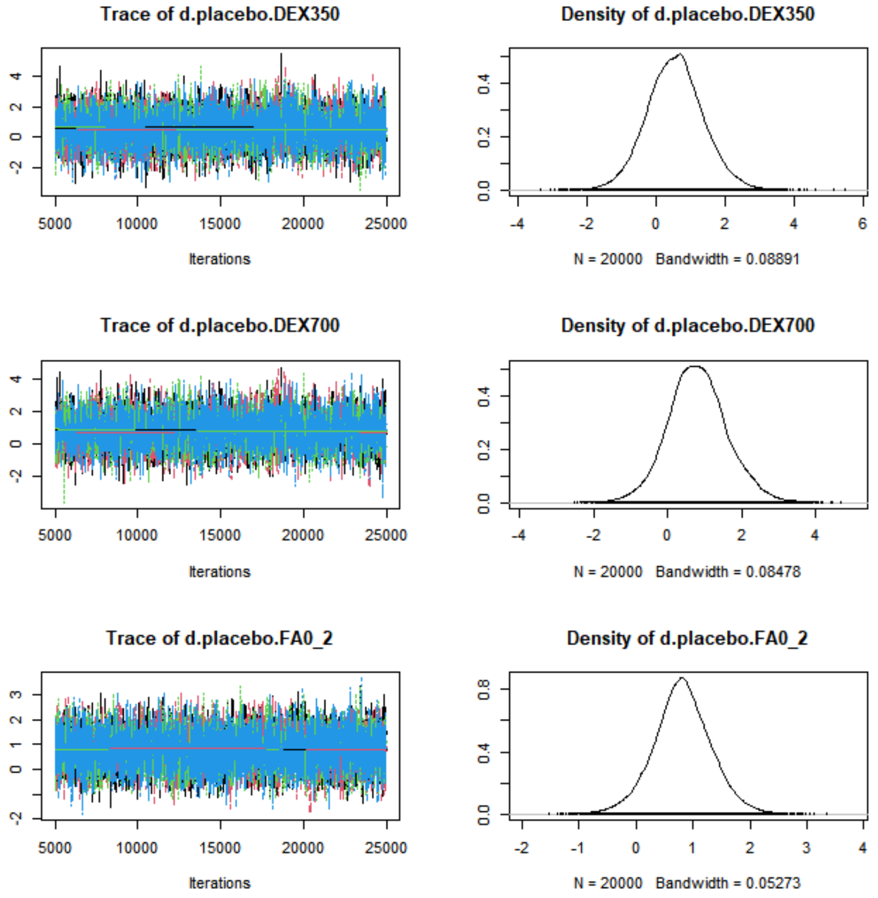

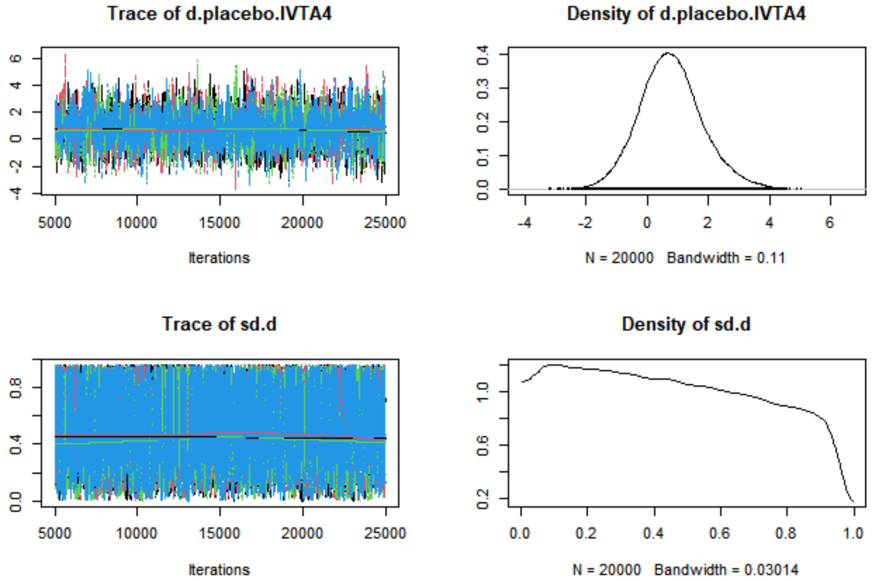

F. IOP rising

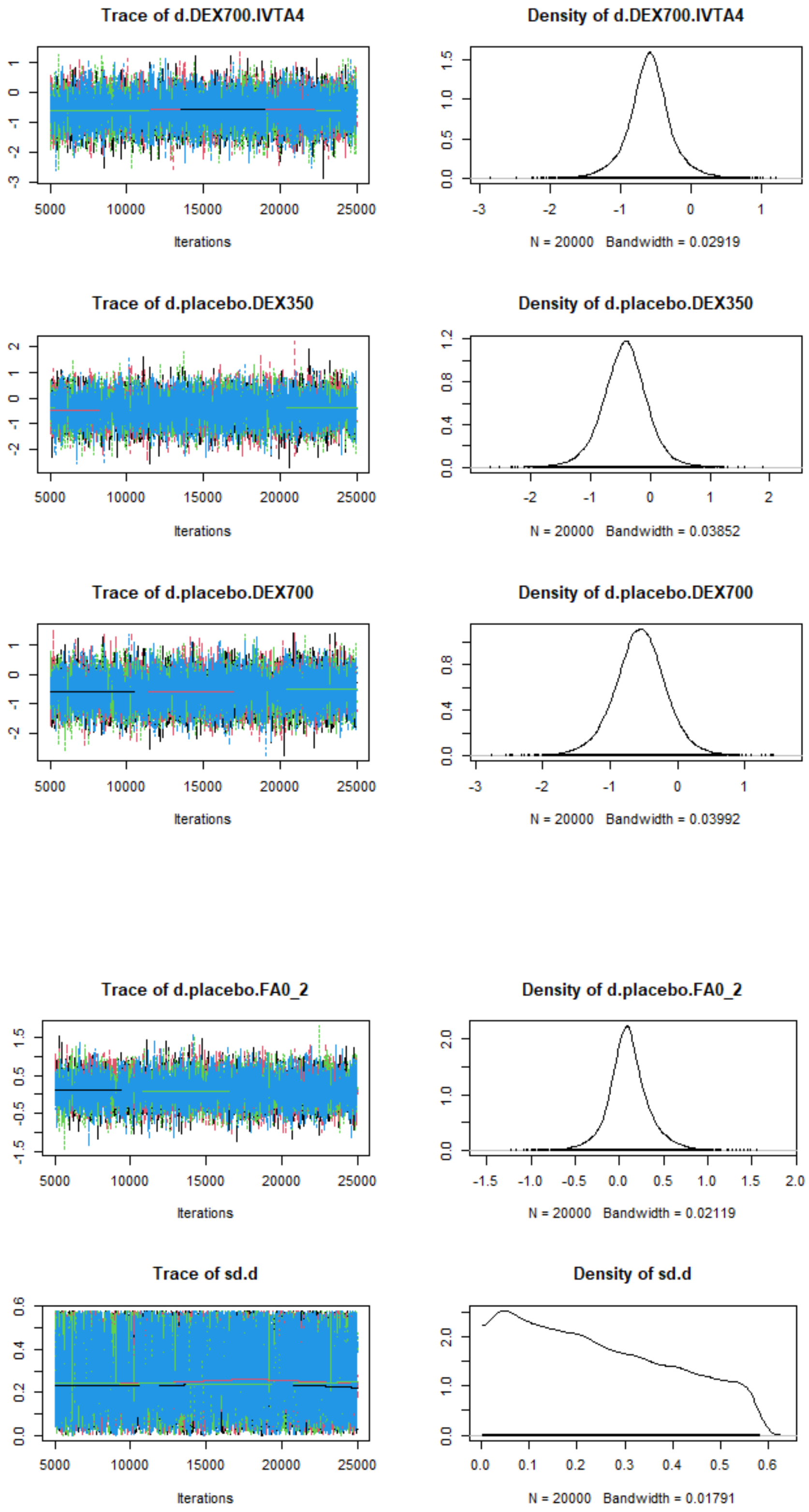
Trace of d.DEX700.IVTA4
Iterations
Density of d.DEX700.IVTA4
N = 20000 Bandwidth = 0.02919
Trace of d.placebo.DEX350
Iterations
Density of d.placebo.DEX350
N = 20000 Bandwidth = 0.03852
Trace of d.placebo.DEX700
Iterations
Density of d.placebo.DEX700
N = 20000 Bandwidth = 0.03992
Trace of d.placebo.FA0_2
Iterations
Density of d.placebo.FA0_2
N = 20000 Bandwidth = 0.02119
Trace of sd.d
Iterations
Density of sd.d
N = 20000 Bandwidth = 0.01791
