# Supplementary material for: Comparative Efficacy and Safety of Advanced Intravitreal Therapeutic Agents for Noninfectious Uveitis: A Systematic Review and Network Meta-Analysis
Source: Front Pharmacol. 2022 Apr 5;13:749312. doi: 10.3389/fphar.2022.749312 (PMC9017745; doi:10.3389/fphar.2022.749312)
Supplement: Supplementary file 4 [file Table2.DOCX]

Supplementary Table S2. Search strategy

| Cochrane Library search strategy: | #1 MeSH descriptor: [Uveitis] explode all trees  #2 uveiti*  #3 MeSH descriptor: [Panuveitis] explode all trees  #4 Panuveitis  #5 MeSH descriptor: [Ophthalmia, Sympathetic] explode all trees  #6 (Ophthalm* near/2 Sympathetic)  #7 MeSH descriptor: [Pars Planitis] explode all trees  #8 Pars Planitis  #9 MeSH descriptor: [Panophthalmitis] explode all trees  #10 Panophthalmiti*  #11 MeSH descriptor: [Uveomeningoencephalitic Syndrome] explode all trees  #12 (Uveomeningoencephaliti* or Vogt Koyanagi Harada or VKH or fuch or Harada disease or harada syndrome or vogt koyanagi disease)  #13 MeSH descriptor: [Behcet Syndrome] explode all trees  #14 (behcet* or triple symptom complex)  #15 MeSH descriptor: [Iridocyclitis] explode all trees  #16 (Iridocycliti* or Heterochromic Cycliti* or anterior scleritis)  #17 MeSH descriptor: [Iritis] explode all trees  #18 Iriti*  #19 Choroiditis  #20 (choroiditi* or retinochoroiditi* or chorioretinitis)  #21 (Blau* syndrome or familial juvenile systemic granulomatosis or Jabs disease)  #22 (Reiter* disease or reiter* syndrome or conjunctivo urethro synovial or urethrooculosynovial syndrome or uroarthritis)  #23 (uveoretinitis or uveo retinitis)  #24 vitritis*  #25 MeSH descriptor: [Retinitis] explode all trees  #26 (retinitis or neuroretinitis)  #27 #1 or #2 or #3 or #4 or #5 or #6 or #7 or #8 or #9 or #10 or #11 or #12 or #13 or #14 or #15 or #16 or #17 or #18 or #19 or #20 or #21 or #22 or #23 or #24 or #25 or #26  #28 MeSH descriptor: [Fluocinolone Acetonide] explode all trees  #29 (Fluocinolone or Fluortriamcinolone or Synalar or Synalar HP or Synalar-HP or Synemol or Synamol or Alvadermo or Capex or Co-Fluocin or Co Fluocin or Cortiespec or Gelidina or Flucinar or Fluocid or Fluodermo or Fluonid or Fluotrex or Flurosyn or Flusolgen or Jellin or Jellisoft or Derma-Smooth FS or Derma Smooth FS or 67-73-2)  #30 MeSH descriptor: [Dexamethasone] explode all trees  #31 (Dexamethasone* or 50-02-2 or Millicorten* or maxidex* or decaspray* or dexpak* or dexasone* or oradexon* or decaject* or hexadecadrol* or hexadrol* or methylfluorprednisolone* or decameth*)  #32 MeSH descriptor: [Triamcinolone Acetonide] explode all trees  #33 (Azmacort* or Kenacort A* or Acetonide-Triamcinolone* or Kenalog* or Kenalog 40* or Tricort40* or Tricort-40* or Tricort 40* or Cinonide)  #34 MeSH descriptor: [bevacizumab] explode all trees  #35 (Mvasi* or Bevacizumab-awwb* or Bevacizumab awwb* or Avastin*)  #36 MeSH descriptor: [[Ranibizumab](" \l "0" \o "Phrase Matches)] explode all trees  #37 ([Ranibizumab](" \l "0" \o "Phrase Matches)* or RhuFab V2*)  #38 MeSH descriptor: [Eylea] explode all trees  #39 MeSH descriptor: [Aflibercept] explode all trees  #40 MeSH descriptor: [Conbercept ] explode all trees  #41 MeSH descriptor: [Brolucizumab] explode all trees  #42 MeSH descriptor: [anti-VEGF] explode all trees  #43 (Polycyclic Compounds or Fused-Ring Compounds or Steroids or Pregnanes or Pregnadienes or Triamcinolone or Triamcinolone Acetonide)  #44 MeSH descriptor: [Drug Implants] explode all trees  #45 MeSH descriptor: [Drug Delivery Systems] explode all trees  #46 (Device* or implant* or shunt* or valve* or tube*)  #47 #28 or #29 or #30 or #31 or #32 or #33 or #34 or #35 or #36 or #37 or #38 or #39 or #40 or #41 or #42 or #43 or #44 or #45 or #46  #48 #27 and #47 |
| --- | --- |
| EMBASE.com search strategy: | ('randomized controlled trial'/exp OR 'randomized controlled trial') AND ('human'/exp OR human) AND ('uveitis'/exp OR uveitis) AND ('fluocinolone acetonide'/exp OR 'fluocinolone acetonide' OR ('intravitreal triamcinolone' OR (intravitreal AND ('triamcinolone'/exp OR triamcinolone) OR 'dexamethasone'/exp OR dexamethasone OR ('intravitreal bevacizumab' OR (intravitreal AND ('bevacizumab'/exp OR bevacizumab) OR 'intravitreal Lucentis' OR (intravitreal AND ('Lucentis'/exp OR Lucentis) OR 'intravitreal Ranibizumab' OR (intravitreal AND ('Ranibizumab'/exp OR Ranibizumab) OR 'intravitreal Eylea' OR (intravitreal AND ('Eylea'/exp OR Eylea) OR 'intravitreal Aflibercept' OR (intravitreal AND ('Aflibercept'/exp OR Aflibercept) OR 'intravitreal Conbercept' OR (intravitreal AND ('Conbercept'/exp OR Conbercept) OR 'intravitreal Brolucizumab' OR (intravitreal AND ('Brolucizumab'/exp OR Brolucizumab) OR 'intravitreal anti-VEGF' OR (intravitreal AND ('anti-VEGF'/exp OR anti-VEGF) OR 'drug delivery system'/exp OR 'drug delivery system' OR (('drug'/exp OR drug) AND ('delivery'/exp OR delivery) AND system) OR 'drug implant'/exp OR 'drug implant') |
| PubMed search strategy: | 1. ((randomized controlled trial[pt]) OR (controlled clinical trial[pt]) OR (randomised[tiab] OR randomized[tiab]) OR (placebo[tiab]) OR (drug therapy[sh]) OR (randomly[tiab]) OR (trial[tiab]) OR (groups[tiab])) NOT (animals[mh] NOT humans[mh])  2. uveiti*[tw] OR Panuveitis[tw] OR (Ophthalm*[tw] AND Sympathetic[tw]) OR Pars Planitis[tw] OR Panophthalmiti*[tw] OR Uveomeningoencephaliti*[tw] OR Vogt Koyanagi Harada[tw] OR VKH[tw] OR fuch[tw] OR Harada disease[tw] OR harada syndrome[tw] OR vogt koyanagi disease[tw] OR behcet*[tw] OR triple symptom complex[tw] OR Iridocycliti*[tw] OR Heterochromic Cycliti*[tw] OR anterior scleritis[tw] OR Iriti*[tw] OR choroiditi*[tw] OR retinochoroiditi*[tw] OR chorioretinitis[tw] OR Blau* syndrome[tw] OR familial juvenile systemic granulomatosis[tw] OR Jabs disease[tw] OR Reiter* disease[tw] OR reiter* syndrome[tw] OR conjunctivo urethro synovial[tw] OR urethrooculosynovial syndrome[tw] OR uroarthritis[tw] OR uveoretinitis[tw] OR uveo retinitis[tw] OR vitritis*[tw] OR retinitis[tw] OR neuroretinitis[tw]  3. Fluocinolone[tw] OR Fluortriamcinolone[tw] OR Synalar[tw] OR Synemol[tw] OR Synamol[tw] OR Alvadermo[tw] OR Capex[tw] OR Co Fluocin[tw] OR Cortiespec[tw] OR Gelidina[tw] OR Flucinar[tw] OR Fluocid[tw] OR Fluodermo[tw] OR Fluonid[tw] OR Fluotrex[tw] OR Flurosyn[tw] OR Flusolgen[tw] OR Jellin[tw] OR Jellisoft[tw] OR Derma Smooth FS[tw] OR 67-73-2[tw] OR Triamcinolone Acetonide*[tw] OR Acetonide, Triamcinolone[tw] OR Cinonide[tw] OR Tricort-40[tw] OR Tricort 40[tw] OR Tricort40[tw] OR Kenalog[tw] OR Kenalog 40[tw] OR Azmacort[tw] OR Kenacort A OR Dexamethasone*[tw] OR 50-02-2[tw] OR Millicorten*[tw] OR maxidex*[tw] OR decaspray*[tw] OR dexpak*[tw] OR dexasone*[tw] OR oradexon*[tw] OR decaject*[tw] OR hexadecadrol*[tw] OR hexadrol*[tw] OR methylfluorprednisolone*[tw] OR decameth*[tw] OR retisert*[tw] OR Device*[tw] OR implant*[tw] OR shunt*[tw] OR valve*[tw] OR tube[tw] OR tubes[tw] OR bevacizumab*[tw] OR Mvasi*[tw] OR Bevacizumab-awwb*[tw] OR Avastin*[tw] OR RhuFab V2*[tw] OR Lucentis*[tw] OR Ranibizumab*[tw] OR Eylea*[tw] OR Aflibercept*[tw] OR Conbercept*[tw] OR Brolucizumab*[tw] OR anti-VEGF*[tw]  4. #2 AND #3  5. #1 AND #4 |
| ClinicalTrials.gov search strategy: | condition: uveitis OR panuveitis OR choroiditis OR pars planitis OR panophthalmitis OR uveomeningoencephalitic OR behcet OR iridocyclitis OR iritis OR retinitis  Intervention: fluocinolone OR dexamethasone OR Triamcinolone Acetonide OR bevacizumab OR Lucentis OR Ranibizumab OR Eylea OR Aflibercept OR Conbercept OR Brolucizumab OR anti-VEGF OR device OR implant OR shunt OR valve OR tube |
