# Supplementary material for: Comparative Efficacy and Safety of Advanced Intravitreal Therapeutic Agents for Noninfectious Uveitis: A Systematic Review and Network Meta-Analysis
Source: Front Pharmacol. 2022 Apr 5;13:749312. doi: 10.3389/fphar.2022.749312 (PMC9017745; doi:10.3389/fphar.2022.749312)
Supplement: Supplementary file 5 [file Table5.docx]

Supplementary Table S5. References of all RCTs included.

| **Callanan 2008** | 1. Callanan DG, Jaffe GJ, Martin DF, Pearson PA, Comstock TL. Treatment of posterior uveitis with a fluocinolone acetonide implant: three-year clinical trial results. Archives of ophthalmology (Chicago, Ill : 1960). 2008;126(9):1191-201. 2. Sheppard JD, Jr., Nguyen QD, Usner DW, Comstock TL. Post-cataract outcomes in patients with noninfectious posterior uveitis treated with the fluocinolone acetonide intravitreal implant. Clinical ophthalmology (Auckland, NZ). 2012;6:79-85. 3. Jaffe GJ, Martin D, Callanan D, Pearson PA, Levy B, Comstock T. Fluocinolone acetonide implant (Retisert) for noninfectious posterior uveitis: thirty-four-week results of a multicenter randomized clinical study. Ophthalmology. 2006;113(6):1020-7. |
| --- | --- |
| **Jaffe 2020** | 1. Callanan D, Nguyen QD, Suhler EB, Paggiarino D, Riedel GE. Reduced risk of recurrence of non-infectious posterior segment uveitis after 0.18 mg fluocinolone acetonide insert: Randomized Trial. Am J Ophthalmol. 2020. 2. Jaffe GJ, Foster CS, Pavesio CE, Paggiarino DA, Riedel GE. Effect of an Injectable Fluocinolone Acetonide Insert on Recurrence Rates in Chronic Noninfectious Uveitis Affecting the Posterior Segment: Twelve-Month Results. Ophthalmology. 2019;126(4):601-10. 3. Jaffe GJ, Pavesio CE: Effect of a Fluocinolone Acetonide Insert on Recurrence Rates in Noninfectious Intermediate, Posterior, or Panuveitis: Three-Year Results. Ophthalmology, 2020 |
| **Kempen 2011** | 1. Kempen JH, Altaweel MM, Holbrook JT, et al. Randomized comparison of systemic anti-inflammatory therapy versus fluocinolone acetonide implant for intermediate, posterior, and panuveitis: the multicenter uveitis steroid treatment trial. Ophthalmology. 2011;118(10):1916-26. 2. Kempen JH, Jabs DA. Benefits of systemic anti-inflammatory therapy versus fluocinolone acetonide intraocular implant for intermediate uveitis, posterior uveitis, and panuveitis : Fifty-four-month results of the Multicenter Uveitis Steroid Treatment (MUST) Trial and Follow-up Study. Ophthalmology. 2015;122(10):1967-75. 3. Kempen JH, Van Natta ML, Altaweel MM, et al. Factors Predicting Visual Acuity Outcome in Intermediate, Posterior, and Panuveitis: The Multicenter Uveitis Steroid Treatment (MUST) Trial. American Journal of Ophthalmology. 2015;160(6):1133-41.e9. 4. Tomkins-Netzer O, Lightman S, Drye L, et al. Outcome of Treatment of Uveitic Macular Edema: The Multicenter Uveitis Steroid Treatment Trial 2-Year Results. Ophthalmology. 2015;122(11):2351-9. 5. Kempen JH, Altaweel MM, Holbrook JT, et al. Association Between Long-Lasting Intravitreous Fluocinolone Acetonide Implant vs Systemic Anti-inflammatory Therapy and Visual Acuity at 7 Years Among Patients With Intermediate, Posterior, or Panuveitis. Jama. 2017;317(19):1993-2005. |
| **Lower 2011** | 1. Lowder C, Belfort R, Jr., Lightman S, et al. Dexamethasone intravitreal implant for noninfectious intermediate or posterior uveitis. Arch Ophthalmol. 2011;129(5):545-53. |
| **NCT02746991 2020** | 1. Medicine and Healthcare Products Regulatory Agency I 190 micrograms intravitreal implant in applicator: summary of product characteristics. 2012. at [https://www.medicines.org.uk/emc/medicine/27636#gref](https://www.medicines.org.uk/emc/medicine/27636#gref).). |
| **Pavesio 2010** | 1. Pavesio C, Zierhut M, Bairi K, Comstock TL, Usner DW. Evaluation of an intravitreal fluocinolone acetonide implant versus standard systemic therapy in noninfectious posterior uveitis. Ophthalmology. 2010;117(3):567-75, 75.e1. |
| **Rahimi 2012** | 1. Rahimi M, Shahrzad SS, Banifatemi M. Comparison of intravitreal injection of bevacizumab and triamcinolone acetonide in the treatment of uveitic macular edema. Iranian journal of immunology : IJI. 2012;9(2):136-44. |
| **Sangwan 2015** | 1. Sangwan VS, Pearson PA, Paul H, Comstock TL. Use of the Fluocinolone Acetonide Intravitreal Implant for the Treatment of Noninfectious Posterior Uveitis: 3-Year Results of a Randomized Clinical Trial in a Predominantly Asian Population. Ophthalmology and therapy. 2015;4(1):1-19. |
| **Shin 2015** | 1. Shin JY, Yu HG. Intravitreal Triamcinolone Injection for Uveitic Macular Edema: A Randomized Clinical Study. Ocul Immunol Inflamm. 2015;23(6):430-6. |
| **Soheilian 2010** | 1. Soheilian M, Rabbanikhah Z, Ramezani A, Kiavash V, Yaseri M, Peyman GA. Bevacizumab vs. triamcinolone. Ophthalmology. 2010;117(4):855-.e2. 2. Soheilian M, Rabbanikhah Z, Ramezani A, Kiavash V, Yaseri M, Peyman GA. Intravitreal bevacizumab versus triamcinolone acetonide for refractory uveitic cystoid macular edema: a randomized pilot study. Journal of ocular pharmacology and therapeutics : the official journal of the Association for Ocular Pharmacology and Therapeutics. 2010;26(2):199-206. |
| **Thorne 2019** | 1. Thorne JE, Sugar EA, Holbrook JT, et al. Periocular Triamcinolone vs. Intravitreal Triamcinolone vs. Intravitreal Dexamethasone Implant for the Treatment of Uveitic Macular Edema: The PeriOcular vs. INTravitreal corticosteroids for uveitic macular edema (POINT) Trial. Ophthalmology. 2019;126(2):283-95. |
| **Staurenghi 2018** | 1. Staurenghi G, Lai TYY, Mitchell P, Wolf S, Wenzel A, Li J, et al. Efficacy and Safety of Ranibizumab 0.5 mg for the Treatment of Macular Edema Resulting from Uncommon Causes: Twelve-Month Findings from PROMETHEUS. Ophthalmology. 2018;125(6):850-62. |
| **Lai 2018** | 1. Lai TYY, Staurenghi G, Lanzetta P, Holz FG, Melissa Liew SH, Desset-Brethes S, et al. EFFICACY AND SAFETY OF RANIBIZUMAB FOR THE TREATMENT OF CHOROIDAL NEOVASCULARIZATION DUE TO UNCOMMON CAUSE: Twelve-Month Results of the MINERVA Study. Retina (Philadelphia, Pa). 2018;38(8):1464-77. |
