# Supplementary material for: Comparative Efficacy and Safety of Advanced Intravitreal Therapeutic Agents for Noninfectious Uveitis: A Systematic Review and Network Meta-Analysis
Source: Front Pharmacol. 2022 Apr 5;13:749312. doi: 10.3389/fphar.2022.749312 (PMC9017745; doi:10.3389/fphar.2022.749312)
Supplement: Supplementary file 6 [file Table3.DOCX]

Supplementary Table S3. All network meta-analysis commands used in R

| Package | Commands |
| --- | --- |
| gemtc  rjags | network <-mtc.network(data)  plot(network) |
|  | model <-mtc.model(network, type = "consistency", factor = 2.5, n.chain=4,likelihood="binom",link="log",linearModel="random")  results <-mtc.run(model, n.adapt = 5000, n.iter = 20000, thin = 1,sampler ="rjags") |
|  | model <-mtc.model(network,type = "consistency",n.chain = 3,likelihood="normal",link="identity",linearModel="random")  results <- mtc.run(model, sampler = "rjags", n.adapt = 5000, n.iter = 20000, thin = 1) |
|  | gelman.plot(results) |
|  | forest(relative.effect(results, "placebo"),digits=4) |
|  | windows()  plot(results) |
|  | ranks<- rank.probability(results,preferredDirection=1)  print(ranks)  plot(ranks, beside=TRUE)  write.csv(ranks,"ranks.csv") |
|  | result.anohe <-mtc.anohe(network, n.adapt=10000, n.iter=15000)  summary.anohe <- summary(result.anohe)  plot(summary.anohe, xlim=log(c(0.2, 5)),digits=4)  dev.off()  summary.anohe |
|  | result<-mtc.nodesplit(network)  summary(result)  names(result)  summary(result$d.A.C)  pdf("nodesplit.pdf")  summary.ns <- summary(result)  print(summary.ns)  plot(summary.ns,digits=4) |
