# Supplementary material for: Comparative Efficacy and Safety of Advanced Intravitreal Therapeutic Agents for Noninfectious Uveitis: A Systematic Review and Network Meta-Analysis
Source: Front Pharmacol. 2022 Apr 5;13:749312. doi: 10.3389/fphar.2022.749312 (PMC9017745; doi:10.3389/fphar.2022.749312)
Supplement: Supplementary file 7 [file Table4.docx]

Supplementary Table S4 Characteristics of included studies

| Study | Design | country | Treatment 1 /(eyes) | Treatment 2 /(eyes) | Treatment 3 /(eyes) | Total No. of patients/eyes | Women/Total No. of patients (%) | Mean age | Mean follow-up year |
| --- | --- | --- | --- | --- | --- | --- | --- | --- | --- |
| Lowder *et al*, 2011 | RCT | Multiple | Surgical DEX implant (350 µg)/76 | Surgical DEX implant (700 µg)/77 | Sham group/76 | 229/229 | 145/229 (63.3%) | 44.6 | 26 weeks |
| Jaffe *et al*, 2020 | RCT | Multiple | Surgical FA implant (0.2 µg/day)/87 | Sham group/42 | n.a. | 129/129 | 79/129 (61.2%) | 48.3 | 36 months |
| NCT02746991, 2020 | RCT | India | Surgical FA implant (0.2 µg/day)/101 | Sham group/52 | n.a. | 153/153 | 96/153 (62.7%) | n.a. | 36 months |
| Pavesio *et al*, 2010 | RCT | Multiple | Surgical FA implant (0.59 mg)/72 | Standard  of care/74 | n.a. | 146/146 | 85/146 (58.2%) | 40.4 | 24 month |
| Kempen *et al*, 2011 | RCT | Multiple | Surgical FA implant (0.59 mg)/245 | Standard  of care/234 | n.a. | 255/479 | 192/255 (75%) | 46.3 | 24 month |
| Callanan *et al*, 2008 | RCT | United States | Surgical FA implant (0.59 mg)/110 | Surgical FA implant (2.1 mg)/168 | n.a. | 278/278 | 201/278 (72%) | 43.5 | 36 month |
| Sangwan *et al*, 2015 | RCT | Multiple | Surgical FA implant (0.59 mg)/117 | Surgical FA implant 2.1 mg)/122 | n.a. | 239/239 | 201/278 (72%) | 41.4 | 36 month |
| Rahimi *et al*, 2012 | RCT | Iran | Intravitreal bevacizumab (1.25 mg)/26 | Intravitreal triamcinolone acetonide (4 mg)/29 | n.a. | 55/60 | 29/55 (53%) | 23.1 | 6 month |
| Soheilian *et al*, 2010 | RCT | Iran | Intravitreal bevacizumab (1.25 mg)/15 | Intravitreal triamcinolone acetonide (2 mg)/16 | n.a. | 31/31 | 17/31 (54.8) | 38.8 | 8 month |
| Thorne *et al*, 2019 | RCT | Multiple | Periocular triamcinolon (40 mg)/74 | Intravitreal triamcinolone acetonide (4 mg)/82 | Dexamethasone intravitreal Implant (700 µg)/79 | 192/235 | 119/192 (62%) | 55.3 | 24 months |
| Shin *et al*, 2015 | RCT | Korea | Intravitreal triamcinolone acetonide (4 mg)/25 | Sham group/25 | n.a. | 50/50 | 54.0 | 52.0 | 6 months |
| Staurenghi *et al*, 2018 | RCT | Multiple | Intravitreal ranibizumab (0.5 mg)/14 | Sham group/7 | n.a. | 21/21 | n.a. | n.a. | n.a. |
| Lai *et al*, 2018 | RCT | Multiple | Intravitreal ranibizumab (0.5 mg)/18 | Sham group/10 | n.a. | 21/21 | n.a. | n.a. | n.a. |

Abbreviations: BCVA, best-corrected visual acuity; DEX, dexamethasone; FA, fluocinolone acetonide; RCT, randomized controlled trial; n.a., not available or not applicable.
