# Supplementary material for: Comparative Efficacy and Safety of Advanced Intravitreal Therapeutic Agents for Noninfectious Uveitis: A Systematic Review and Network Meta-Analysis
Source: Front Pharmacol. 2022 Apr 5;13:749312. doi: 10.3389/fphar.2022.749312 (PMC9017745; doi:10.3389/fphar.2022.749312)
Supplement: Supplementary file 8 [file Image1.PDF]

Supplementary Figure S1. Gelman diagnostic statistics

Gelman diagnostic statistic values for all model parameters are close to 1 indicating that every Markov Monte Carlo chains converged well.

A. BCVA improvement

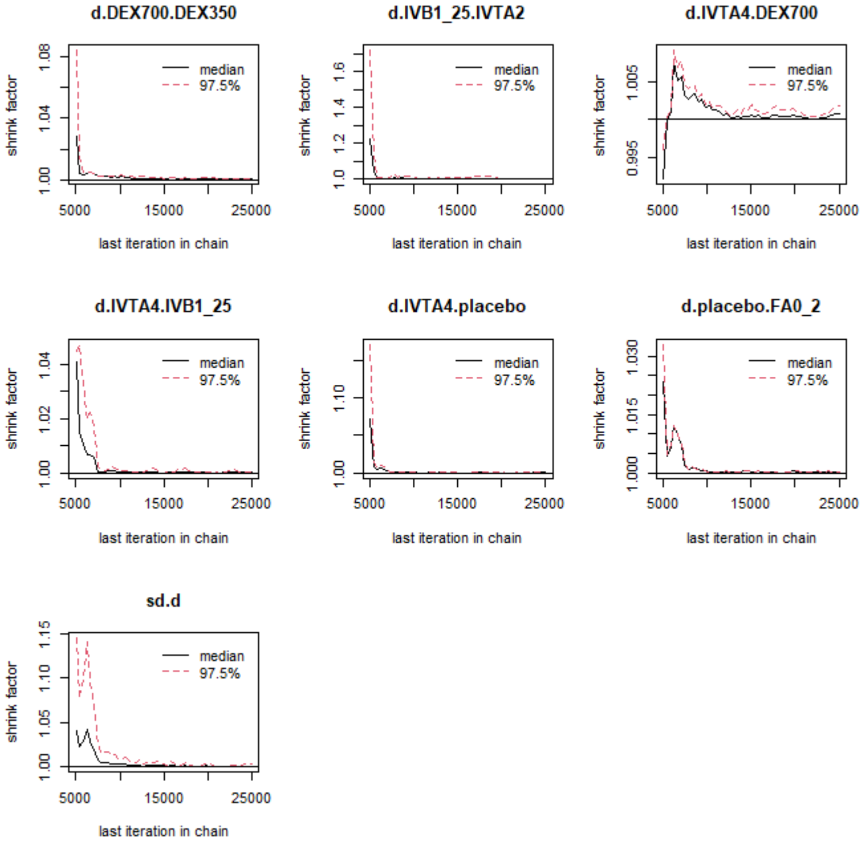

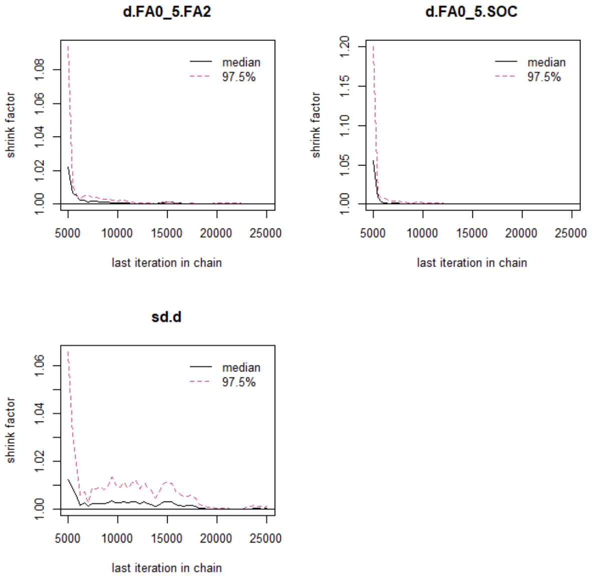

B. Vitreous haze improvement

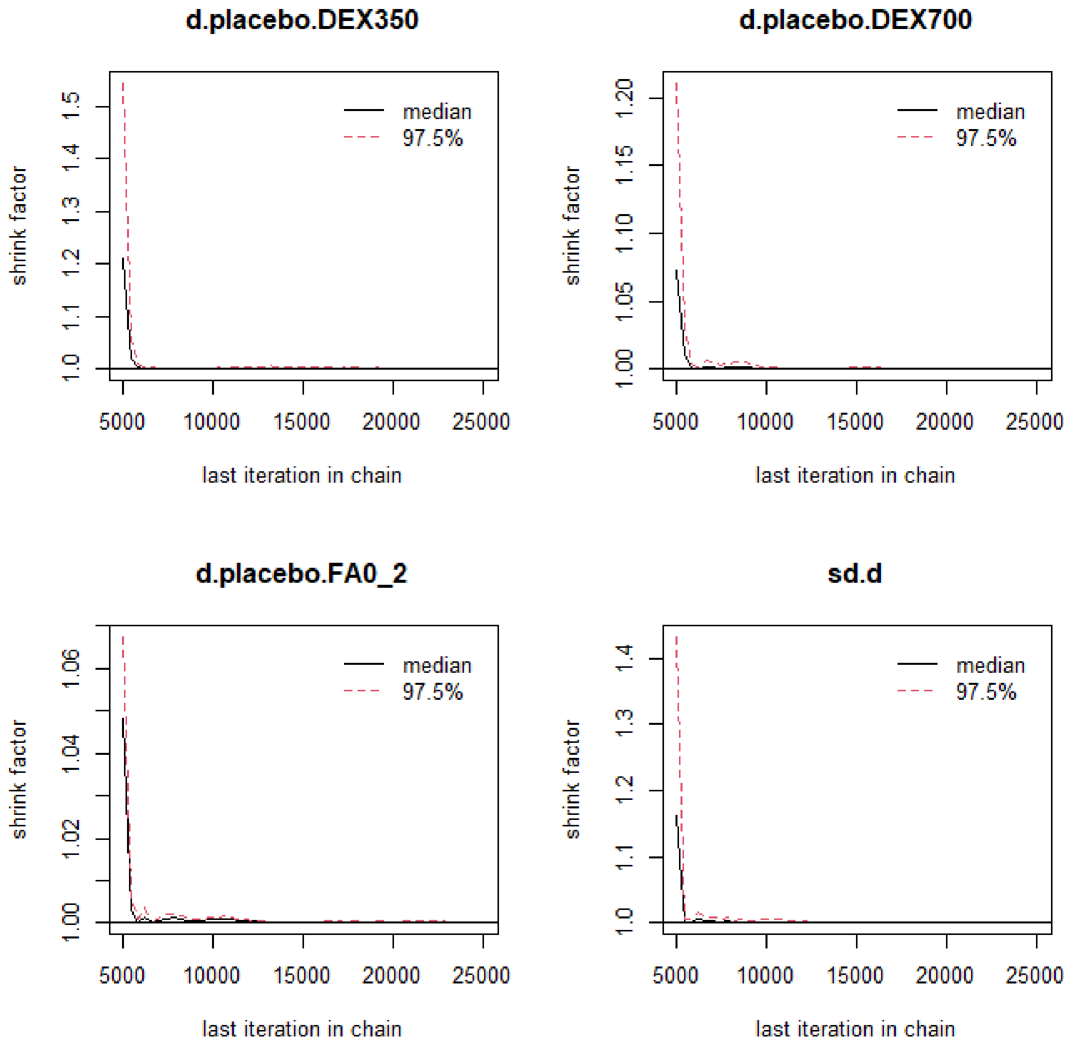

C. Uveitis recurrence

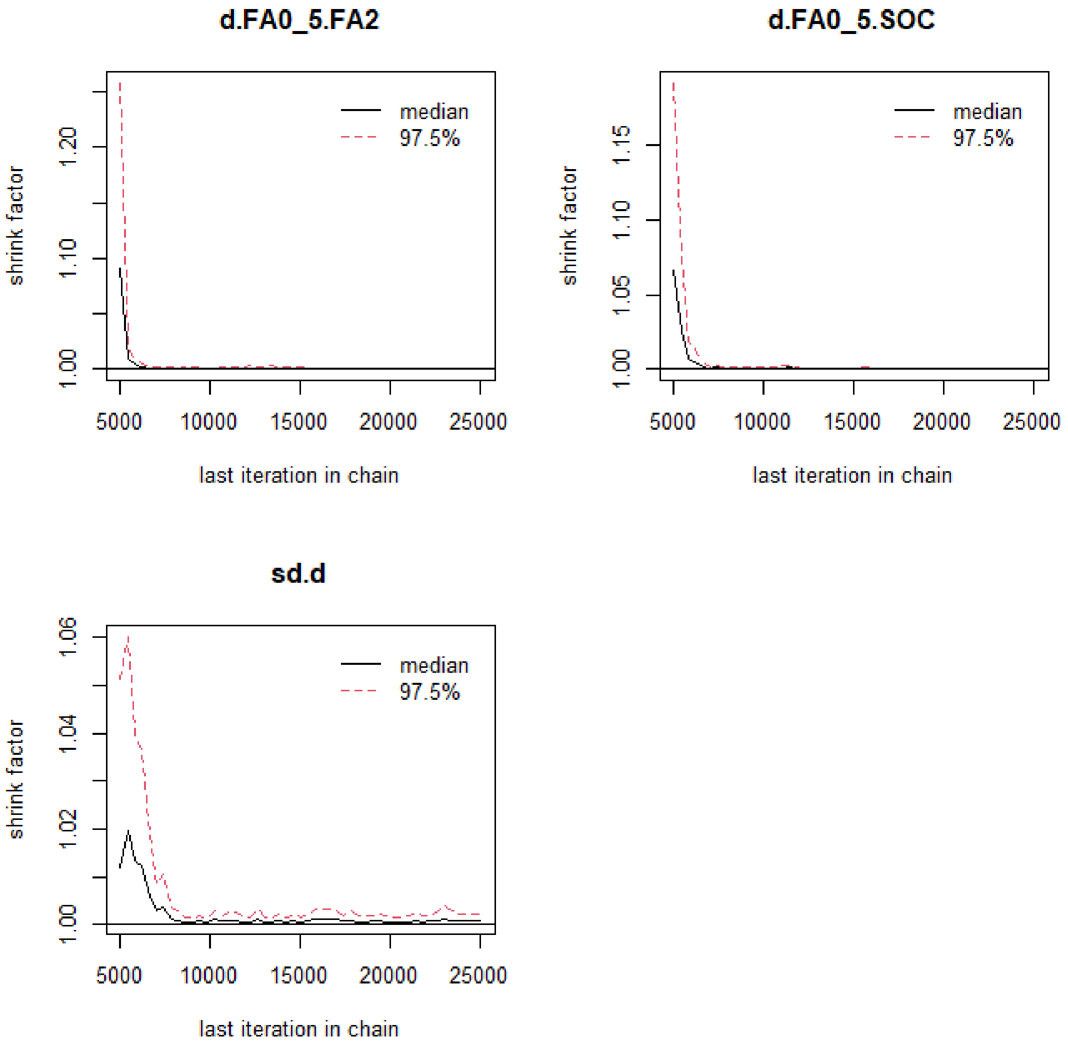

D. Change of macular retinal thickness

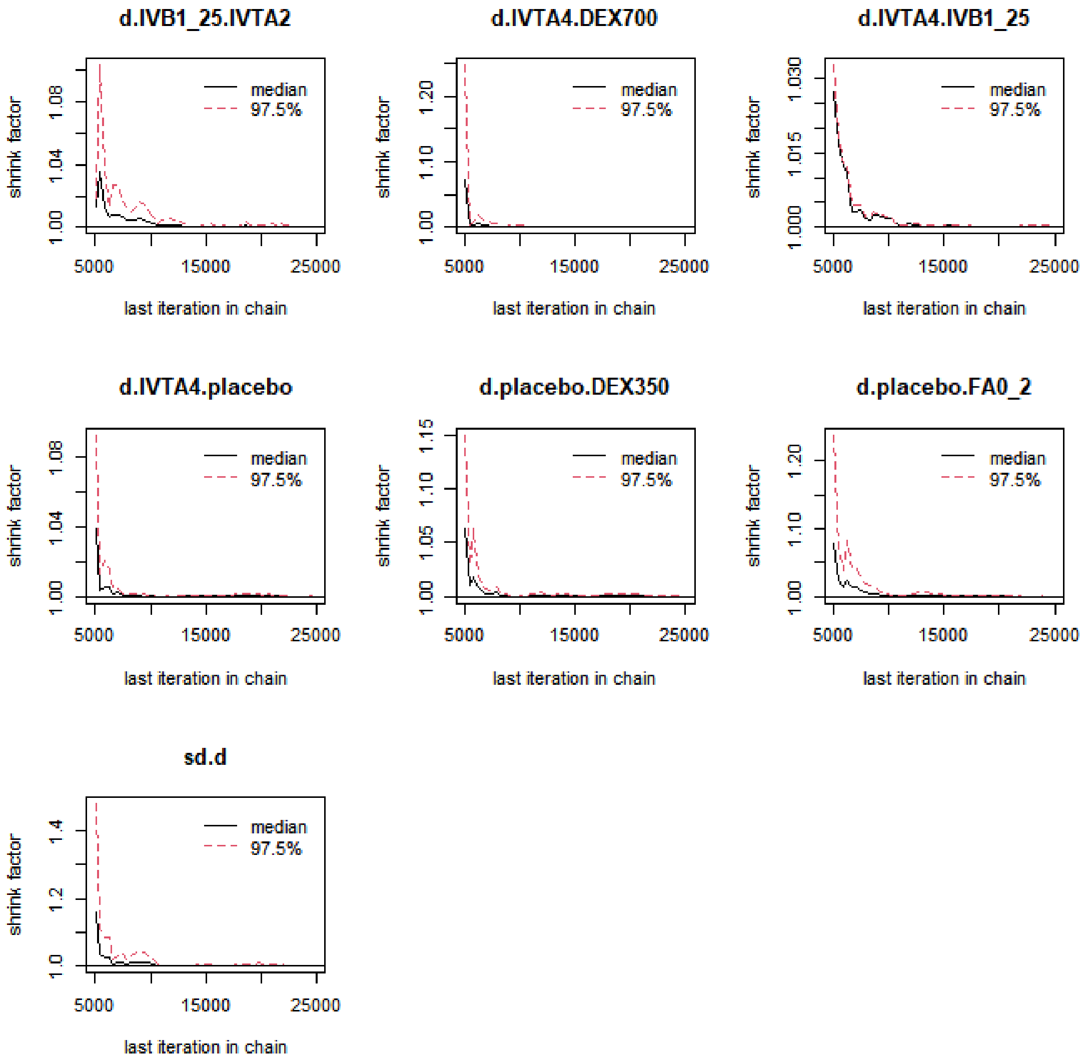

E. Incidence of cataract

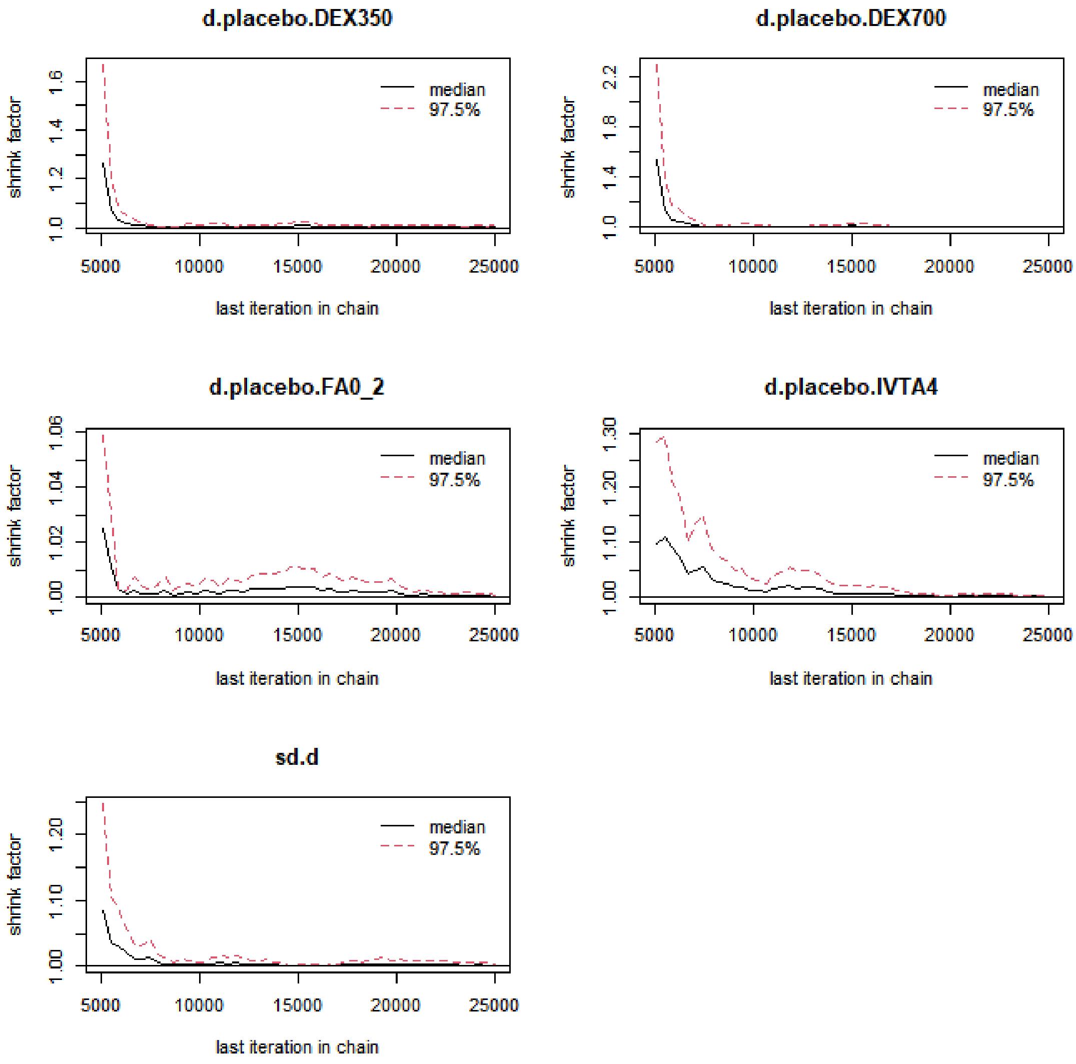

F. IOP rising

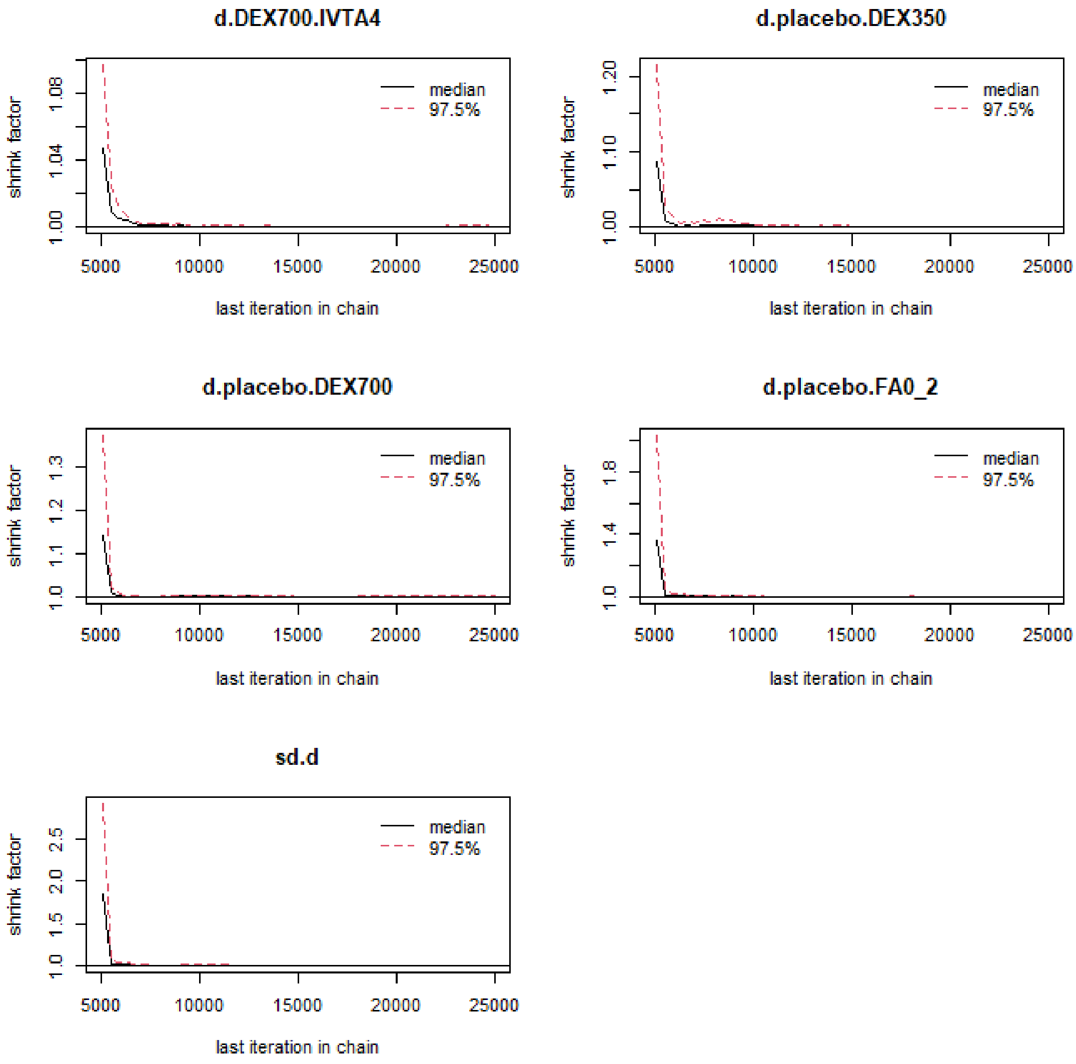
d.DEX700.IVTA4
shrink factor
1.00
1.04
1.08
median
97.5%
5000
10000
15000
20000
25000
last iteration in chain
d.placebo.DEX350
shrink factor
1.00
1.10
1.20
median
97.5%
5000
10000
15000
20000
25000
last iteration in chain
d.placebo.DEX700
shrink factor
1.0
1.1
1.2
1.3
median
97.5%
5000
10000
15000
20000
25000
last iteration in chain
d.placebo.FA0_2
shrink factor
1.0
1.4
1.8
median
97.5%
5000
10000
15000
20000
25000
last iteration in chain
sd.d
shrink factor
1.0
1.5
2.0
2.5
median
97.5%
5000
10000
15000
20000
25000
last iteration in chain
